# Activation of the oncogenic transcription factor B-Myb via multisite phosphorylation and prolyl *cis/trans* isomerization

**DOI:** 10.1093/nar/gky935

**Published:** 2018-10-13

**Authors:** Eugen Werwein, Hannah Cibis, Daniel Hess, Karl-Heinz Klempnauer

**Affiliations:** 1 Institute for Biochemistry Westfälische-Wilhelms-Universität, D-48149 Münster, Germany; 2 Friedrich Miescher Institute for Biomedical Research, Maulbeerstr. 66, CH-4058 Basel, Switzerland

## Abstract

The oncogenic transcription factor B-Myb is an essential regulator of late cell cycle genes whose activation by phosphorylation is still poorly understood. We describe a stepwise phosphorylation mechanism of B-Myb, which involves sequential phosphorylations mediated by cyclin-dependent kinase (Cdk) and Polo-like kinase 1 (Plk1) and Pin1-facilitated peptidyl-prolyl *cis/trans* isomerization. Our data suggest a model in which initial Cdk-dependent phosphorylation of B-Myb enables subsequent Pin1 binding and Pin1-induced conformational changes of B-Myb. This, in turn, initiates further phosphorylation of Cdk-phosphosites, enabling Plk1 docking and subsequent Plk1-mediated phosphorylation of B-Myb to finally allow B-Myb to stimulate transcription of late cell cycle genes. Our observations reveal novel mechanistic hierarchies of B-Myb phosphorylation and activation and uncover regulatory principles that might also apply to other Myb family members. Strikingly, overexpression of B-Myb and of factors mediating its activation strongly correlates with adverse prognoses for tumor patients, emphasizing B-Myb's role in tumorigenesis.

## INTRODUCTION

Progression through the eukaryotic cell cycle requires sequential activation and deactivation of various proteins and genes. Recently, the DREAM complex (DP, RB-like, E2F4 and MuvB (synMuv genes, class B)) was recognized as a master coordinator of cell cycle-dependent gene expression (1). The mammalian DREAM complex consists of the MuvB core complex and the repressor proteins DP1, E2F4 and p130(RBL2) and occupies promoters of cell cycle genes during quiescence or after a p53-induced cell cycle arrest, thereby inhibiting their transcription (2–5). Upon cell cycle entry, Cdk-mediated phosphorylation of p130 leads to disassembly of the DREAM complex allowing expression of G1/S-phase genes (6–8). In S-phase, the MuvB complex associates with transcription factor B-Myb to form the Myb-MuvB (MMB) complex, which then activates G2/M-phase genes, either directly or through recruitment of transcription factor FoxM1 (2,3,6,9–11). The exact function of B-Myb within the MMB complex is not yet fully understood.

B-Myb is a member of the Myb proto-oncogene family (12). As the other family members, B-Myb has a highly conserved N-terminal DNA-binding domain (DBD), a transcriptional activation domain (TAD) and a C-terminal negative regulatory domain (NRD). B-Myb is ubiquitously expressed in proliferating cells and is essential for cell proliferation (13,14). The activity of B-Myb is highly regulated on transcriptional and post-transcriptional levels during the cell cycle. B-Myb is transcriptionally repressed in G1, activated by cyclin A/Cdk2-mediated phosphorylation during S-phase and subsequently degraded during late G2 in an ubiquitin-dependent manner (15–18). Besides its role in the MMB complex, B-Myb is thought to perform transcription-independent functions during mitosis through the formation of the Myb-Clafi complex (19). Importantly, how B-Myb switches between transcriptional and non-transcriptional functions is poorly understood.

B-Myb undergoes extensive phosphorylation at approximately 15 Cdk-dependent phosphosites during its activation (20–22). Initial efforts to link phosphorylation of certain sites to specific B-Myb functions have been inconclusive, resulting in the current ‘all-or-nothing’ model of B-Myb activation by phosphorylation. We have recently shown that B-Myb adopts distinct phosphorylation patterns upon DNA damage, which correlates with transcriptional shutdown during recovery time (23). These findings suggest that different functions of B-Myb are modulated by specific phosphorylation patterns, prompting us to investigate the cell cycle-dependent phosphorylation of B-Myb in more detail.

## MATERIALS AND METHODS

### Cell culture, transfection and infection

Human HEK293 and Hela were grown in DMEM with 10% fetal calf serum (FCS). PC3 and HepG2 cells were grown in DMEM/Hams F12 and RPMI1640, respectively, supplemented with 10% FCS. These cell lines were obtained from the American Type Culture Collection. Quail QT6 cells were grown in Iscove's modified DMEM medium supplemented with 8% FCS and 2% chicken serum. Cell lines were maintained at 37°C and 5% CO_2_ and were free of mycoplasma contamination. Transient transfection of plasmid DNAs was performed by calcium phosphate co-precipitation. B-Myb expression was silenced with siRNA duplexes targeting the sequences CUG GAA CUC UAC CAU CAA A (B-myb siRNA_3), GAA ACA UGC UGC GUU UGU A (B-myb siRNA_4). SiRNA targeting Renilla luciferase (AAA CAU GCA GAA AAU GCU G) was used as negative control. SiRNAs (100 nM) were transfected using Metafectene™ Pro (Biontex), according to manufacturer's protocols. Cells were harvested 16–48 h after transfection. Lentiviral expression vectors were co-transfected with the lentiviral packaging plasmids pMD2.G and psPAX2 into HEK293T cells to generate infectious viral particles, followed by infection of target cells and puromycin selection to eliminate uninfected cells.

### Drug treatment and cell cycle synchronization

HepG2 and Hek293 cells were synchronized at G1/S-boundary by treatment with 4 mM thymidine for 20 h followed by release for 10 h and then re-treatment with 4 mM thymidine for 20 h (double thymidine block). For S-phase enrichment HepG2 cells were treated with 4 mM thymidine for 20 h and then released for 1 h. For synchronization in the G2/M-phase HepG2 or Hek293 cells were treated with 10 μM RO-3306 (Santa Cruz Biotechnology) for 18 h and released for 30 min, with 0.5 μg/ml nocodazole (Sigma-Aldrich) for 8 h or with 5 μM S-trityl-l-cysteine (Santa Cruz Biotechnology) for 12 h. For inhibitor treatment, the Cdk inhibitors roscovitine (Santa Cruz Biotechnology) and RO-3306 or Plk1 inhibitor, BI2536 were added to the cells for 30–45 min at 25, 10 and 0.1 μM, respectively.

### Expression vectors

Expression vectors for human B-Myb wt and siRNA-resistant B-Myb mutants were described previously (24). B-Myb point mutants T444A, T476A, 2SA (S335A, S339A) and CBM (R695A, L697A) were constructed by PCR using appropriate oligonucleotides to introduce the mutations. B-Myb 10AP (T266A, S282A, S393, T405, S452, S494, T515, T518, T520, S577) and B-Myb 6SA (T286, S287, S317, S335, S339, S343) mutants were obtained through gene synthesis and subcloned in pcDNA4 vector. B-Myb 2AP (T266A, S282A), 3AP (S393, T405, S452), 5AP (T266A, S282A, S393, T405, S452) and 5BP (S494, T515, T518, T520, S577) mutants were generated by subcloning B-Myb sequences from plasmid pcDNA4+huB-Myb(10AP). Myc-B-Myb was generated by PCR and cloned into pcDNA4. peGFP-B-Myb encodes a fusion protein of eGFP and full-length human B-Myb fused C-terminally to GFP. Truncated derivatives of human B-Myb encoding amino acids 1–207, 206–700, 206–509, 272–509, 1–207 plus 485–700, 466–700, 485–700, 1–509, 206–335, 335–509 fused to eGFP or GST were generated by cloning the corresponding parts of the B-Myb coding region into peGFP-C1, 2 or 3 and pGEX-4T-1, 2 or 3 utilizing appropriate restriction sites. Lentiviral vectors for Myc-B-Myb wt, 10AP and 6SA mutants were generated by replacing the dsRed coding region of pLVX-dsRed-Monomer-C1 (Clontech) by the coding sequence of B-Myb wt, 10AP and 6SA. The expression vectors for Myc-tagged human Plk1 (25) and GST-tagged human Plk1, GST-PBD(wt) and GST-PBD(FAA) (26) were obtained from B.Schermer and I.Hoffmann. pRC/cyclin A2, pCMV+Cdk2(wt) and pCMV+Cdk2(dn) have been described (17). GFP- and GST-fused cyclin A2 were generated by subcloning the coding region and into peGFP-C1 and pGEX-4T-1. Expression vectors for wt or C113A Pin1 were obtained from Ferrari (27) and Hofmann (28).

### Recombinant protein expression and purification

GST-tagged proteins were purified essentially as described (24). In brief, protein expression was performed in *Escherichia coli* Rosetta™(DE3) bacteria, which were lysed in GST lysis buffer (50 mM Tris–HCl, pH 8,0, 150 mM NaCl, 1% (v/v) Triton X-100, 1 mM DTT and 1 mM PMSF) for 30 min on ice, sonicated and centrifuged at 14 000 × g for 1 h to obtain extracts containing soluble proteins. To purify GST-fused proteins appropriate amounts of extracts were added to glutathione sepharose 4B beads (GE Healthcare), incubated at 4°C for 1–3 h and washed three times with ELB buffer. Bound proteins were analyzed by SDS-PAGE or used otherwise.

### Co-immunoprecipitation, GFP-Trap^®^ and GST-pulldown experiments

Cells were lysed in ELB buffer (50 mM Tris–HCl pH 7,5, 120 mM NaCl, 20 mM NaF, 1 mM EDTA, 6 mM EGTA, 15 mM sodium pyrophosphate, 0.5 mM Na_3_VO_4_, 1 mM phenylmethylsulfonyl fluoride and 0.2% NP-40) for 15 min on ice. Lysates were centrifuged at 14 000 × g for 20 min and the supernatant was used as whole cell extract. Aliquots of the whole cell extract were immunoprecipitated with the appropriate antibodies for 3–12 h at 4°C. 25 μl Protein-A/G sepharose beads (GE Healthcare, 50% slurry) were then added and incubated further for 3–5 h at 4°C under constant agitation. Immune complexes bound to the beads were collected by centrifugation and washed 3–5 times with ELB buffer. Bound proteins were analyzed by western blotting or used for *in vitro* kinase assay. For Pin1-IP cells were lysed in modified ELB buffer (50 mM Tris–HCl pH 8, 150 mM NaCl, 20 mM NaF, 1 mM EDTA, 6 mM EGTA, 15 mM sodium pyrophosphate, 0,5 mM Na_3_VO_4_, 1 mM phenylmethylsulfonyl fluoride and 0,5% Triton X-100). Immunoprecipitations of B-Myb were performed with rabbit antisera raised against the N-terminal (‘#53’) or the C-terminal regions (‘brian’) of mouse B-Myb (17). Pin1, Plk1, cyclin A and Cdk2 were immunoprecipitated with antibodies as described below. Unrelated rabbit or mouse IgGs (Santa Cruz Biotechnolgy, sc-2027 or sc-2025) were used in control-IPs. For GFP-Trap^®^ experiments QT6 cells transfected with expression vectors for GFP and GFP fusion proteins were lysed in ELB buffer 24 h after transfection. Aliquots of whole cell extracts were then incubated with 10 μl GFP-Trap^®^ agarose beads (Chromotec, München) for 3–5 h at 4°C. Beads were washed 3–5 times with ELB buffer. Bound proteins were analyzed by western blotting, used for *in vitro* kinase- assay or for mass spectrometry analysis. For GST pulldown assays, glutathione sepharose beads loaded with the appropriate amounts (1–5 μg) of GST or GST-fusion proteins were incubated with whole cells extracts for 1–12 h at 4°C. The beads were washed 3–5 times with ELB buffer. Bound proteins were analyzed by western blotting or used for *in vitro* kinase-assay.

### Western blotting, Far-WB analysis and antibodies

Whole cell extracts and purified proteins were boiled in 1× SDS-PAGE sample buffer and run on 10–12% SDS-PAGE gels. Resolved proteins were transferred to nitrocellulose membranes followed by immunostaining with the appropriate antibodies. For Far-Western blotting proteins transferred to membrane were incubated in renaturing buffer (100 mM NaCl, 0.5 mM EDTA, 20 mM Tris–HCl pH 7,6, 10% glycerol, 0,2% NP 40, 1 mM DTT and 2% (w/v) skim milk at 4°C for 6 h. Soluble, GST-fused proteins were added to the membrane, which was then incubated at 4°C overnight, washed twice with PBS + 0.05%Tween-20 and subjected to standard immunostaining. Primary antibodies for immunostaining were as follows: mouse anti-B-Myb (Lx015.1, 1:10) (9), mouse anti-Myb-DBD (5E11, 1:10) (17), rabbit anti-phospho-B-Myb(T487) (Abcam, ab76009, 1:3 000–10 000), mouse anti β-actin (Sigma-Aldrich, AC-15, 1:10 000), mouse anti-GFP (Sigma-Aldrich, 7.1 + 13.1, 1:5000), mouse anti-Plk1 (Santa Cruz Biotechnology, F-8, 1:1000–5000), mouse anti-Pin1 (Santa Cruz Biotechnology, G-8, 1:500–5000), rabbit anti-cyclin A (Santa Cruz Biotechnology, H-432, 1:500), mouse anti-Cdk2 (Santa Cruz Biotechnology, D-12, 1:1000), mouse anti-Myc-tag (9E10, 1:500–5000), mouse anti-HA.11-tag (6B12, 1:5000), rabbit anti-phospho-PLK binding motif (SpTP) (Cell Signaling Technology, D73F6, 1:3000), anti-histone H3 (Abcam, ab1791, 1:1000), rabbit anti-GST (i11) (24).

### *In vitro* kinase and phosphatase assay

*In vitro* protein kinase assays were performed as described (23). Briefly, Myc-Plk1 or GFP-cyclin A/Cdk2 were immunoprecipitated with anti-Myc antibodies or GFP-Trap beads from QT6 cells transfected with the relevant expression vectors. As control, an identical immunoprecipitation was performed with untransfected cells. Endogenous cyclin A/Cdk2 complexes were immunoprecipitated with anti-cyclin A or Cdk2 antibodies. GST proteins were purified from bacterial extracts by binding to glutathione sepharose beads. Immunoprecipitates and bound GST-proteins were washed three times each with ELB buffer and kinase buffer (25 mM Tris–HCl, pH 7.5, 10 mM MgCl_2_, 5 mM beta-glycerophosphate, 2 mM DTT, 100 μM Na_3_VO_4_), mixed together and incubated for 30 min at 30°C in kinase buffer containing 50 μM ATP and 4 μCi of γ-^32^P-ATP per reaction (specific activity 3000 Ci/mmol). Plk1 inhibitor, BI 2536, and Cdk inhibitor, roscovitine, were added to the reaction mix at 100 nM and 25 μM, respectively. The reactions were stopped by adding 2× SDS-PAGE sample buffer and analyzed by SDS-PAGE and autoradiography. Kinase assays with bacterially expressed GST-Plk1 were performed with GST-Plk1 bound to glutathione sepharose beads. *In vitro* phosphorylated GST-B-Myb fragments to be used in GST-pulldown, far-WB or WB analysis were obtained similarly, except that γ-^32^P-ATP was excluded from reaction mix and the reaction was stopped either by washing the beads twice with ELB buffer or by adding 2× SDS-PAGE sample buffer. *In vitro* phosphatase assays were performed with the whole cell extracts prepared by boiling the cells in FastAP phosphatase buffer containing 0.5% SDS for 10 min, followed by 10-fold dilution with FastAP phosphatase buffer and then dephosphorylated with 10 units FastAP phosphatase (Fermentas) for 1 h at 37°C. The reactions were stopped by adding 2× SDS-PAGE sample buffer.

### Flow cytometry

For cell cycle analysis cells were trypsinized, fixed with 70% ice-cold ethanol in PBS for 1 h at −20°C, washed with PBS (+0.5% BSA) and stained with propidium iodide (50 μg/ml PI and 25 μg/ml RNase A in PBS) for 1 h at room temperature. For quantification of mitotic index cells were fixed as above, washed twice with PBS (+0.5% BSA) and incubated for 30 min with anti-phospho-histone H3(S10) Alexa Fluor^®^ 488 conjugate (Cell Signaling Technology, D2C8, 1:100) at room temperature, followed by washing with PBS (+0.5% BSA) and PI staining. FACS analysis was performed using a Beckman-Coulter Cytomics FC500 flow cytometer. 10 000–15 000 cells were counted per condition in every experiment.

### PRECOG and gene regulation analysis

Meta *Z*-scores were obtained from web-based database PRECOG (https://precog.stanford.edu) (29). Web-based resource TargetGeneReg (http://www.targetgenereg.org) was used to classify genes as targets of DREAM/MMB regulation (30).

### Limited proteolysis

GFP-B-Myb was transiently expressed together with or without Pin1 in QT6 cells and immunoprecipitated with GFP-Trap beads. Beads were washed three times each with modified RIPA buffer (50 mM Tris–HCl pH 7.5, 500 mM NaCl, 50 mM NaF, 2 mM EDTA, 0,5 mM Na_3_VO_4_, 1 mM phenylmethylsulfonyl fluoride, 0.5% Na-deoxycholate, 0.1% SDS and 0.5% NP-40) and PBS. Trypsin (0.05 ng/μl final concentration in PBS) was added for 5–15 min at 18–30°C. The reactions were stopped by addition of 3× SDS-PAGE sample buffer, immediately followed by boiling for 10 min. and analyzed by 10% SDS-PAGE and western blotting.

### Immunostaining and confocal microscopy

Cells cultivated on coverslips were fixed with 4% paraformaldehyde in PBS for 10 min, followed by incubation with 50 mM NH_4_Cl for 10 min at room temperature. The cells were then permeabilized with PBS containing 0.2% NP40 for 10 min, blocked with 5% bovine serum albumin (BSA) in PBS and immunostained with primary antibodies in PBS + 5% BSA. Alexa Fluor^®^ 488-coupled anti-rabbit (Invitrogen, A11034, 1:2000) immunoglobulins were used as secondary antibodies. Nuclei were stained with DAPI (10 μg/ml) at room temperature. Microscopic images were acquired with Leica TCS SL or Leica TCS SP8 confocal system using the Leica confocal software (Leica Microsystems) and with Leica DM IRB microscope.

### Reverse transcription quantitative PCR (RT-qPCR)

Total cellular RNA was isolated with the Nucleo Spin^®^ RNA II Kit (Macherey-Nagel), as suggested by the manufacturer. Total RNA (500 ng) was reverse-transcribed with the SuperScript VILO Master Mix Kit (Thermo Fisher Scientific) in a total volume of 20 μl according to the manufacturer's instructions. RT-qPCR reactions were done in 96-well plates using the StepOnePlus RT-PCR instrument (Applied Biosystems) following the sample maximization method (31). Each reaction was performed as triplicate in a 20 μl volume containing 1× Power SYBR Green PCR Master Mix (Applied Biosystems), 50 nM of each primer and 0.2 μl cDNA. The cycling conditions were as follows: 95°C for 10 min, followed by 40 cycles of 95°C for 15 s and 60°C for 60 s. Each experiment included a no-template control. PCR reaction specificity was confirmed by DNA melting curve analysis and gel electrophoresis of products. A standard curve for each gene was generated using serial 10-fold dilutions of mixed cDNAs and the PCR reaction efficiencies were determined by calibration curve according to the formula *E* = 10^(−1/slope)^. The following primers were used: Cdk1: 5′- CATGGCTACCACTTGACCTGT-3′ and 5′-AAGCCGGGATCTACCATACC-3′, CycB1: 5′-CAGATGTTTCCATTGGGCTT-3′ and 5′- TACCTATGCTGGTGCCAGTG-3′, Plk1: 5′-GGCAACCTTTTCCTGAATGA-3′ and 5′-TCCCACACAGGGTCCTTCTTC-3′, TBP: 5′- ATCCTCATGATTACCGCAGC-3′ and 5′-GAGAGTTCTGGGATTGTACCG-3′. Relative gene expression was calculated according to the 2^−ΔΔCq^ (quantification cycle) method.

### Mass spectrometry analysis

The protein spots were excised from the gel, reduced with 10 mM TCEP, alkylated with 20 mM iodoacetamide and cleaved with either 0.1 μg porcine sequencing grade trypsin (Promega) for 16 h or first with 0.1 μg LysC (WAKO) for 6 h followed by a 16 h digestion with 0.1 μg AspN (Roche) in 25 mM ammonium bicarbonate (pH 8.0) at 37°C. The extracted peptides were analyzed by capillary liquid chromatography tandem mass spectrometry with an EASY-nLC 1000 using the two-column set up (Thermo Scientific). The peptides were loaded in 0.1% formic acid, 2% acetonitrile in water onto a peptide trap (Acclaim PepMap 100, 75 μm × 2 cm, C18, 3 μm, 100 Å) at a constant pressure of 600 bar. Then they were separated, at a flow rate of 150 nl/min with a linear gradient of 2–6% buffer B in buffer A in 3 min followed by an linear increase from 6 to 22% in 40 min, 22–28% in 9 min, 28–36% in 8 min, 36–80% in 1 min and the column was finally washed for 12 min at 80% B (buffer A: 0.1% formic acid in water, buffer B: 0.1% formic acid in acetonitrile) on a 50 μm × 15 cm ES801 C18, 2 μm, 100 Å column mounted on a DPV ion source (New Objective) connected to a Orbitrap Fusion (Thermo Scientific). The data were acquired using 120 000 resolution for the peptide measurements in the Orbitrap with a top T (3 s) method. All samples were analyzed twice. First using HCD fragmentation of the precursors and fragment measurement in the ion trap according to the recommendations of the manufacturer (Thermo Scientific) and secondly with a combination of consecutive CID and HCD fragmentations both measured eachin the ion trap and additionally in the Orbitrap with 30 000 resolution. Mascot Distiller 2.5 was used to combine corresponding HCD and the CID spectra for individual precursors and MASCOT 2.5 searching swissprot version 2015_01 or a custom DB including the sequence GFP_B-Myb was used to identify the peptides. Tryptic digestes were searched with tryptic specificity allowing for up to three incomplete cleavage sites. For the combined LysC/AspN digests the specificity was set to N-terminal cleavage at Asp and Glu and C-terminal cleavage at Lys with up to 5 missed cleavages. Carbamidomethylation of cysteine (+57.0245) was set as a fixed modification, phosphorylation of serine, threonine and tyrosine (+79.9663 Da) oxidation of methionine (+15.9949 Da) and acetylation of protein N-termini (+42.0106 Da) were set as variable modifications. Parent ion mass tolerance was set to 5 ppm and fragment ion mass tolerance to 0.6 Da. The results were combined and validated with the program Scaffold Version 4.4 and ScaffoldPTM 2.2.0 (Proteome Software, Portland, USA). Peptide identifications were accepted if they could be established at >50.0% probability as specified by the Peptide Prophet algorithm (32,33) and phosphorylation sites were accepted if they had a >50% site probability as calculated with ScaffoldPTM.

### Statistical analysis and data availability

Statistical analysis was performed using a two-tailed, unpaired *t* test. Values are means ± standard deviations. Web-based *t* test calculator from GraphPad Software was used for analysis of statistical significance. The experiments were not randomized and the investigators were not blinded to allocation during experiments and outcome assessment. Western blot, immunoprecipitation and immunofluorescence data presented are representative of at least 3 independent experiments.

## RESULTS

### Pin1 stimulates B-Myb phosphorylation by inducing conformational changes

**Figure 1.**
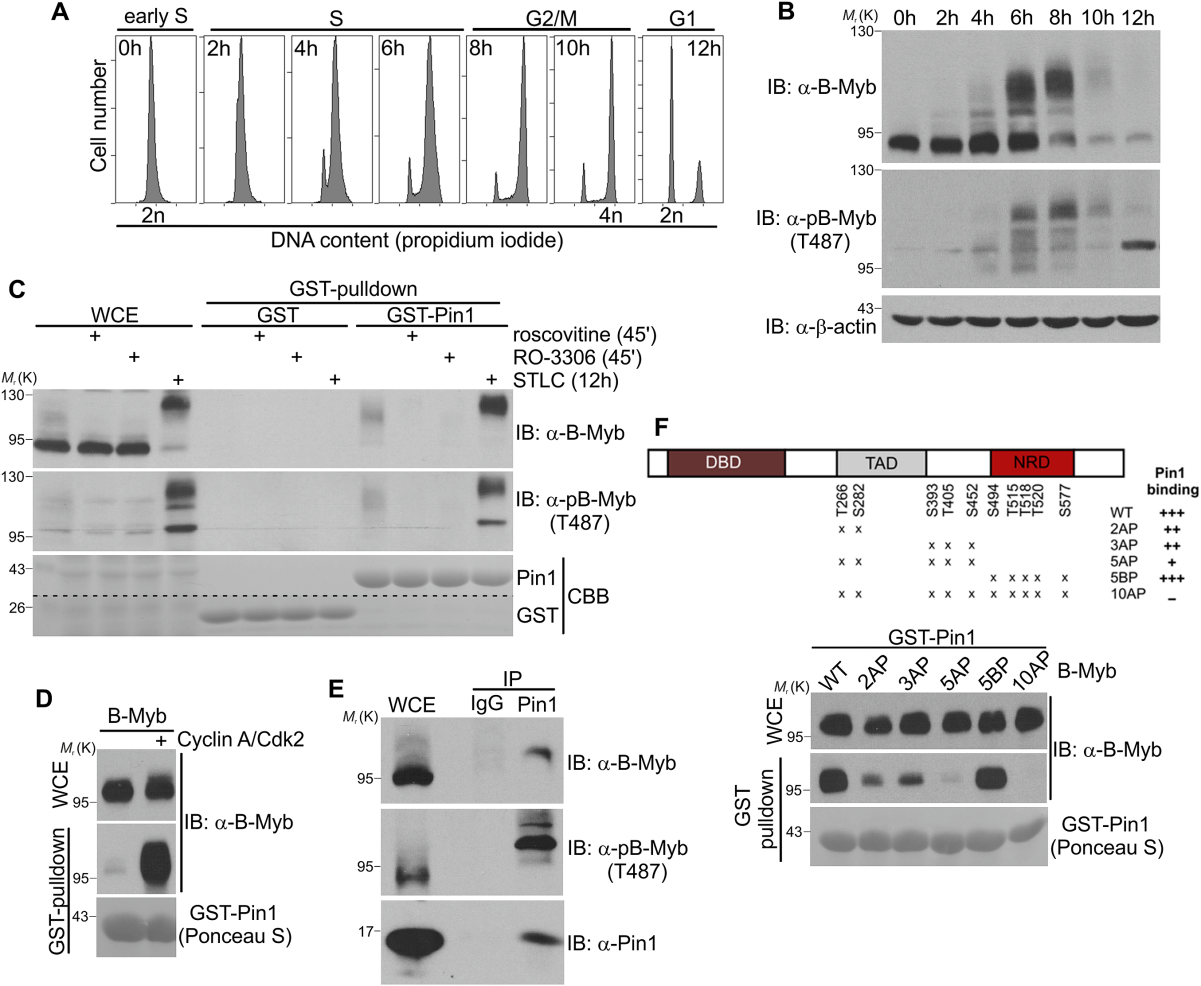
Pin1 interacts with B-Myb in a Cdk-phosphorylation-dependent manner. (**A**) HepG2 cells were synchronized by double thymidine block at the G1/S-boundary and released as indicated. Cell cycle progression was monitored by propidium iodide staining and flow cytometry. (**B**) Extracts from the cells used in A were analyzed by western blotting (WB) with the indicated antibodies. (**C**) Lysates of Hek293 cells pretreated with or without roscovitine or RO-3306 for 30 min or treated with STLC for 12 h were incubated with glutathione-sepharose beads loaded with GST or GST-Pin1. Bound proteins and aliquots of the whole cell extracts (WCE) were analyzed by western blotting with antibodies against B-Myb and phospho-B-Myb(T487). Loading of the beads with GST-proteins was confirmed by Coomassie Brilliant Blue (CBB) staining of the blot. (**D**) Lysates of QT6 cells transiently expressing B-Myb and cyclin A/Cdk2 were incubated with glutathione-sepharose beads loaded with GST-Pin1. Bound proteins and WCE were analyzed by Ponceau S staining and WB. (**E**) Lysates of HepG2 cells were immunoprecipitated with Pin1-specific or control IgG antibodies. Immunoprecipitates and WCE were analyzed by WB with antibodies against B-Myb, phospho-B-Myb(T487) or Pin1. (**F**) *Top*: Schematic representation of human B-Myb with alanine substitutions at indicated Cdk2-phosphosites. DBD: DNA binding domain, TAD: transactivation domain, NRD: negative regulatory domain. Numbers refer to amino acids. *Bottom*: Lysates of QT6 cells transiently expressing the indicated B-Myb proteins were incubated with glutathione-sepharose beads loaded with GST-Pin1. Bound proteins and WCE were analyzed by Ponceau S staining and WB.

We first examined the cell cycle-dependent dynamics of B-Myb phosphorylation using HepG2 cells synchronized by double thymidine block at the beginning of the S-phase and released into the cell cycle for different times. Western blot analysis with a monoclonal antibody against B-Myb showed that B-Myb is strongly expressed in S-phase and is extensively modified in late S/G2-phase as evidenced by a strong electrophoretic mobility shift (Figure 1A and B). We also used a phospho-specific antibody, which has previously been shown to specifically recognize B-Myb phosphorylated at T487 (10,23,34). Phosphorylation at T487 was barely detectable early in the S-phase and increased upon release culminating in hyper-phosphorylated B-Myb forms in the late S/G2-phase.

Besides T487, B-Myb is phosphorylated by cyclin-dependent kinases at a number of Ser or Thr residues followed by Pro (20–22). Such sites, when phosphorylated, can serve as binding sites for the prolyl-peptidyl cis/trans isomerase Pin1 (35). Pin1 translates specific phosphorylation marks into changed protein conformation by isomerization of the pSer/pThr-Pro bond and, thus, can alter the function of its target proteins by inducing changes in their conformation. This prompted us to study whether Pin1 plays a role in B-Myb phosphorylation, given its well-known function as a molecular switch to control the function of its clients, especially of a number of mitosis-relevant proteins (35–39).

To explore a role of Pin1 in B-Myb phosphorylation we first examined whether B-Myb and Pin1 interact with each other. Endogenous B-Myb from Hek293 or HepG2 cell extracts was readily pulled down by GST-Pin1 (Figure 1C and Supplementary Figure S1A). Importantly, Pin1 specifically interacted with slower migrating, phosphorylated forms of B-Myb, which was abolished in extracts from cells pretreated with broad Cdk inhibitor roscovitine or more selective RO-3306, which preferentially inhibits cyclin A-associated Cdk1/Cdk2 kinases (40). Inhibitors were added shortly before cell lysis to prevent any cell cycle alterations as confirmed by flow cytometry (Supplementary Figure S1B). Conversely, the interaction between B-Myb and GST-Pin1 was strongly enhanced in extracts from mitotic cells which were obtained by treatment with the Eg5 kinesin inhibitor, S-trityl-L-cysteine (STLC) (Figure 1C and Supplementary Figure S1A). STLC treatment arrests cells in early mitosis leading to accumulation of hyper-phosphorylated B-Myb as evidenced by strong mobility shift and increased T487 phosphorylation (Figure 1C and Supplementary Figure S1A). Also, ectopic coexpression of cyclin A/Cdk2 and B-Myb strongly stimulated B-Myb binding to GST-Pin1 (Figure 1D). Far western blot analysis with recombinant GST-Pin1 and an *in vitro* phosphorylated GST-B-Myb fragment containing most of the known Cdk2 phosphosites proved that Pin1 bound directly to B-Myb (Supplementary Figure S1C). Finally, immunoprecipitation of extracts from HepG2 cells with Pin1-specific antibodies demonstrated interaction of the endogenous proteins (Figure 1E). Again, Pin1-antibodies specifically coprecipitated slower migrating, phosphorylated forms of endogenous B-Myb. Note that the slow-mobility forms of B-Myb that appear at the late S/G2 phase are less abundant in the input sample because we used un-synchronized cells for the co-precipitation experiment.

To gain further insight into B-Myb/Pin1 interaction we used a series of B-Myb mutants lacking previously identified Cdk phosphorylation sites and subjected them to GST-Pin1 pulldown experiments. This demonstrated that loss of such phosphosites decreased the association with Pin1, culminating in the B-Myb(10AP) mutant which lacks 10 Cdk phosphosites and shows no interaction with Pin1 (Figure 1F). We concluded that Pin1 and B-Myb interact *in vivo* and *in vitro* in a phosphorylation-dependent manner.

**Figure 2.**
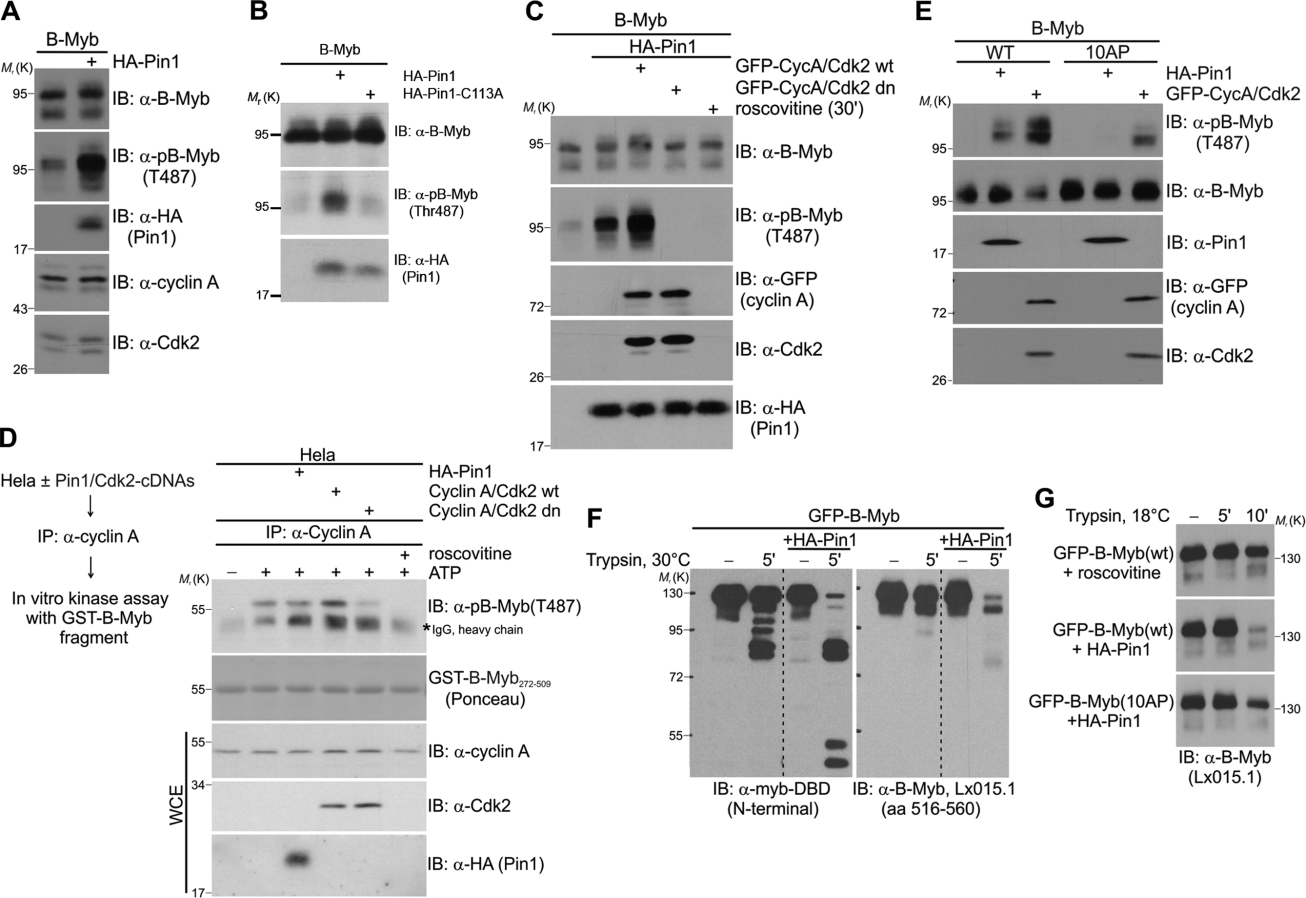
Pin1 facilitates Cdk-dependent phosphorylation and induces conformational changes in B-Myb. (**A–C**) Whole cell extracts from Hela cells transiently transfected with indicated expression vectors were analyzed by WB. Cdk2 (dn) is a catalytically inactive, dominant-negative mutant of Cdk2. Roscovitine was added to the cells 30 min prior to the lysis. Pin1-C113A is a catalytically inactive mutant of Pin1. (**D**) *In vitro* kinase assay with cyclin A/Cdk2 complexes immunoprecipitated from untransfected or transfected Hela cells and recombinant GST-B-Myb (aa 272–509) fragment. Roscovitine was added to the reaction mix to demonstrate specificity of the assay. (**E)** Whole cell extracts from QT6 cells transiently transfected with indicated expression vectors were analyzed by WB. (**F–G**) Limited proteolysis assay. Lysates prepared from QT6 cells transiently expressing GFP-B-Myb together with or without HA-Pin1 were immunoprecipitated with GFP-trap beads. The immunoprecipitates were then subjected to proteolysis with limiting amounts of trypsin at 18 or 30°C for the indicated times and analyzed by western blotting with different B-Myb-specific antibodies. Roscovitine was added to the cells 30 min before lysis.

Next, we analyzed whether Pin1 influences the B-Myb phosphorylation using phosphorylation of T487 as a readout. Ectopically co-expressed wild-type Pin1 strongly stimulated phosphorylation of B-Myb on T487 (Figure 2A) whereas the catalytically inactive Pin1-mutant C113A failed to increase the phosphorylation (Figure 1B). Importantly, the stimulatory effect of wild-type Pin1 was further facilitated by cyclin A/Cdk2 coexpression and completely abrogated by dominant-negative Cdk2 mutant (dn) or roscovitine, confirming that Pin1-stimulated T487 phosphorylation was indeed mediated by a cyclin-dependent kinase (Figure 2C). We then asked whether Pin1 stimulates B-Myb phosphorylation indirectly through modulating cyclin A/Cdk2 activity, that was previously shown to phosphorylate B-Myb (15–17), or whether Pin1 acts directly through modification of B-Myb protein itself. Therefore, we assessed the protein amounts and the kinase activity of cyclin A/Cdk2 complexes upon ectopic expression of Pin1. As shown in Figure 2A, the protein amounts of cyclin A/Cdk2 remained unchanged upon Pin1 expression. Furthermore, *in vitro* phosphorylation of a recombinant GST-B-Myb fragment with cyclin A/Cdk2 complexes immunoprecipitated via cyclin A from untransfected or Pin1-transfected cells revealed that their kinase activity is not enhanced by Pin1 (Figure 2D). As a further control, we also performed kinase assays with extracts from cells transfected with cyclin A/Cdk2 wt or dn mutant, showing increased or decreased kinase activity, respectively. Importantly, *in vitro* phosphorylation of B-Myb was completely abrogated by roscovitine, further demonstrating the specificity of the assay (see lanes on the right side of Figure 2D). Also, under conditions when ectopically expressed Pin1 or cyclin A/Cdk2 stimulated B-Myb phosphorylation *in vivo*, we did not observe any changes in the cell cycle distribution between untransfected and transfected cells, ruling out that the Pin1-induced phosphorylation was an indirect effect due to altered cell cycle distribution (Supplementary Figure S2). Thus, we concluded that Pin1 most likely facilitates phosphorylation of B-Myb directly through inducing conformational changes of B-Myb, as shown for several mitotic proteins before.

To examine this possibility further, we first analyzed the ability of Pin1 to stimulate phosphorylation of T487 in the B-Myb(10AP) mutant, which shows no interaction with Pin1 (as shown in Figure 1F) but, importantly, still contains the intact T487 phosphosite. We found that Pin1 failed to stimulate T487 phosphorylation in B-Myb(10AP) demonstrating that Pin1 binding to B-Myb is required to facilitate B-Myb phosphorylation at T487 (Figure 2E). Notably, cyclin A/Cdk2 also phosphorylated B-Myb(10AP) less efficiently at T487 than B-Myb(wt) indicating that T487 is a poorer substrate for cyclin A/Cdk2 in B-Myb(10AP) than in B-Myb(wt). A possible reason for this observation could be a step-wise or hierarchical order of phosphorylation where the phosphorylation of an ‘early’ phosphosite (which is absent in B-Myb(10AP)) recruits Pin1 to catalyze conformational changes in B-Myb which then facilitates phosphorylation of ‘late’ phosphosites (like T487). This would also be consistent with the observation that the catalytically inactive Pin1 mutant failed to increase phosphorylation at T487 (Figure 2B). To examine if Pin1 induces conformational changes in B-Myb we performed limited proteolysis experiments with purified GFP-B-Myb expressed with or without Pin1. Proteolysis occurs preferentially at accessible, conformationally flexible protein regions, whereas rigid regions remain degradation-resistant, thus permitting to monitor local unfolding (41). In line with the idea of Pin1-induced conformational changes, B-Myb co-expressed with Pin1 underwent faster cleavage than B-Myb expressed alone as demonstrated by using two different B-Myb antibodies against epitopes located in the N- or C-terminal regions of B-Myb, suggesting Pin1-induced prolyl isomerization in B-Myb (Figure 2F). Moreover, the B-Myb(10AP) mutant co-expressed with Pin1 showed less pronounced degradation as compared to B-Myb(wt) demonstrating that Pin1 binding to B-Myb is required to facilitate conformational changes in B-Myb (compare middle and bottom panels in Figure 2G). GFP-B-Myb isolated from cells pretreated with roscovitine and used as a marker of unphosphorylated B-Myb protein was degradation-resistant under these conditions, as expected (Figure 2G, upper panel). Altogether, the limited proteolysis data indicate that B-Myb undergoes phosphorylation-dependent conformational changes, which are catalyzed by Pin1.

### B-Myb interacts with cyclin A through a C-terminal Cy motif

**Figure 3.**
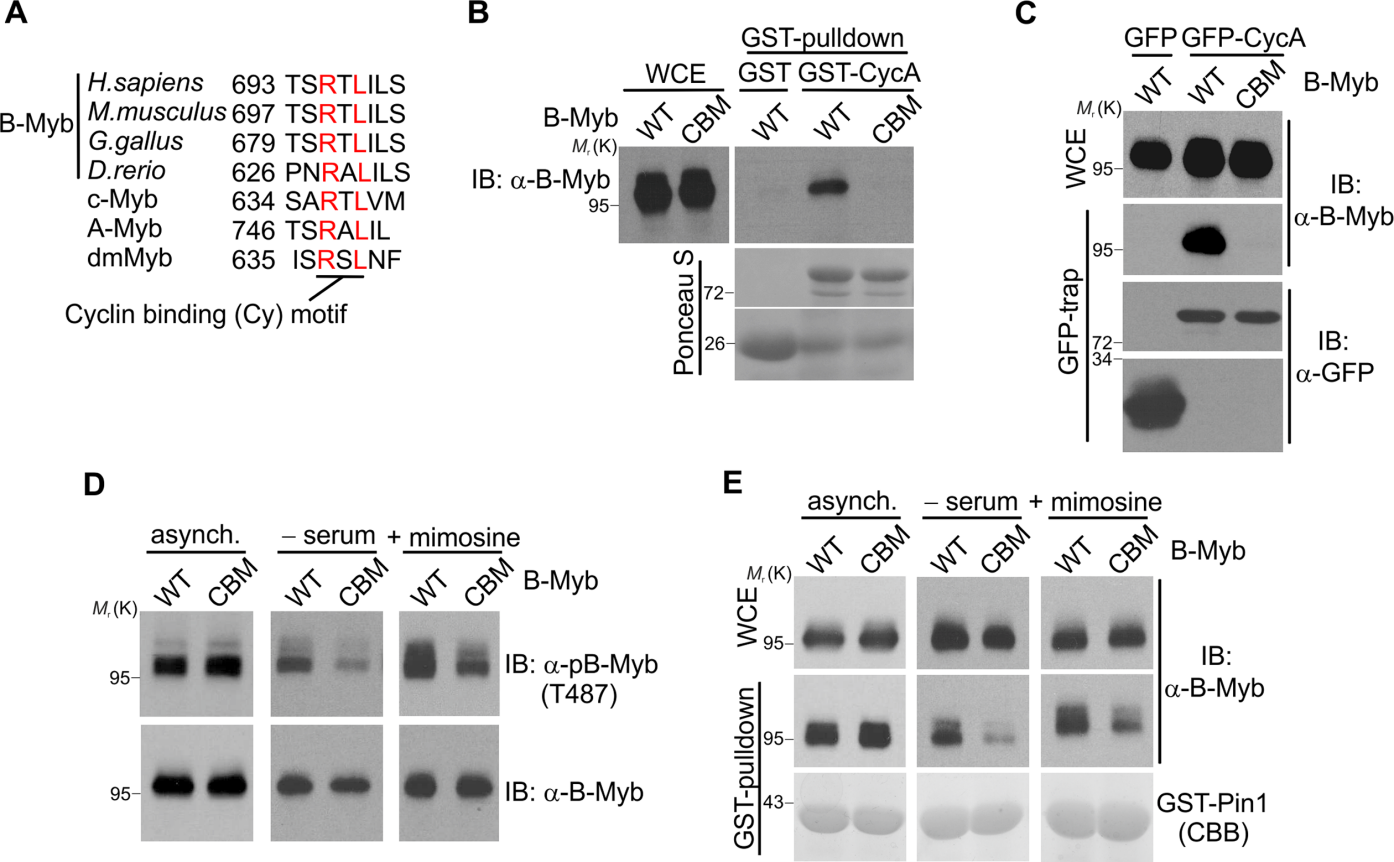
B-Myb interacts with cyclin A through a C-terminal Cy motif. (**A**) Amino acid sequence alignment of the conserved Cy motif in B-Myb from different species, human c-Myb and A-Myb and dmMyb from *D. melanogaster*. The numbers refer to the first amino acid shown. (**B**) Lysates of QT6 cells transiently expressing B-Myb(wt) or B-Myb(CBM) were incubated with glutathione-sepharose beads loaded with GST or GST-cyclin A. Bound proteins and WCE were analyzed by western blotting with antibodies against B-Myb. Loading of the beads with GST-proteins was confirmed by Ponceau S staining of the blot. (**C**) QT6 cells were transiently transfected with expression vectors for B-Myb(wt), B-Myb(CBM), GFP or GFP-cyclin A, as indicated. Cell lysates were immunoprecipitated with GFP-trap beads. Immunoprecipitates and WCE were analyzed by WB. (**D–E**) QT6 cells transiently expressing B-Myb(wt) or B-Myb(CBM) were cultivated as asynchronous population (asynch.) or were incubated in serum-free or mimosine-containing medium for 24 h (-serum/+mimosine). In panel D, whole cell extracts were analyzed by WB as indicated. In panel E, cell extracts were subjected to GST-pulldown with GST-Pin1, followed by WB. Loading of the beads with GST-Pin1 was confirmed by CBB staining.

Given the role of cyclin A-dependent kinase in B-Myb phosphorylation surprisingly little is known about its interaction with B-Myb. Cyclin A/Cdk2 bind their substrates via bipartite recognition motifs with Cdk2 directly targeting phosphoacceptor sites (consensus S/T-P-x-R/K) and cyclin binding to distal Cy (R-x-L) motifs (42–44). Studies in yeast suggested that Cy motifs in Cdk substrates provide selective advantage over substrates lacking them (45). We were therefore interested to examine B-Myb for a functional cyclin binding motif. The B-Myb sequence contains several potential Cy motifs with one highly conserved motif at the extreme C-terminus (Figure 3A). We disrupted this motif in the B-Myb(CBM) mutant (cyclin-binding mutated) by replacing the highly conserved R695 and L697 with alanine to obtain a protein that failed to interact with cyclin A *in vitro* or *in vivo* (Figure 3B–C). We then examined the influence of the CBM mutation on the phosphorylation of B-Myb. We used pulldown with GST-Pin1 as a measure of the overall phosphorylation of Cdk-dependent phosphosites (Figure 3D) as well as phosphorylation-specific antibodies to monitor phosphorylation at T487 (Figure 3E). Surprisingly, there was no difference in overall phosphorylation or phospho-T487 between wild-type B-Myb and the CBM-mutant in asynchronous growing cells. However, Cy motifs have recently been implicated in the fine-tuning of substrate sensitivity towards Cdk activity, thereby contributing to the timing of a substrate's phosphorylation during the cell cycle and promoting the phosphorylation under the low Cdk levels (46). Thus, we reasoned that the Cy motif in B-Myb might be particularly important when Cdk activity is low (e.g. in the late G1 or early S-phase). To test this idea we enriched the cells expressing B-Myb(wt) or the CBM mutant in the G1-phase by short-term serum starvation or by mimosine treatment. Flow cytometry confirmed the enrichment (Supplementary Figure S3). Under these conditions the CBM mutant was indeed phosphorylated less efficiently than B-Myb(wt) as judged by the level of T487 phosphorylation and the reduced binding of B-Myb(CBM) to GST-Pin1 as a readout of overall B-Myb's SP/TP-phosphorylations (Figure 3D–E, middle and right panels). These results suggest that the Cy motif in B-Myb might be particularly important under low cyclin A/Cdk levels.

Overall, our data suggest that Cdk-dependent phosphorylation of B-Myb is not only regulated through the level of Cdk activity but is also modulated via the Cy motif and the Pin1-inducible accessibility of specific phosphosites through conformational changes.

### T476 phosphorylation mediates interaction of B-Myb with Plk1 in the G2/M phase

**Figure 4.**
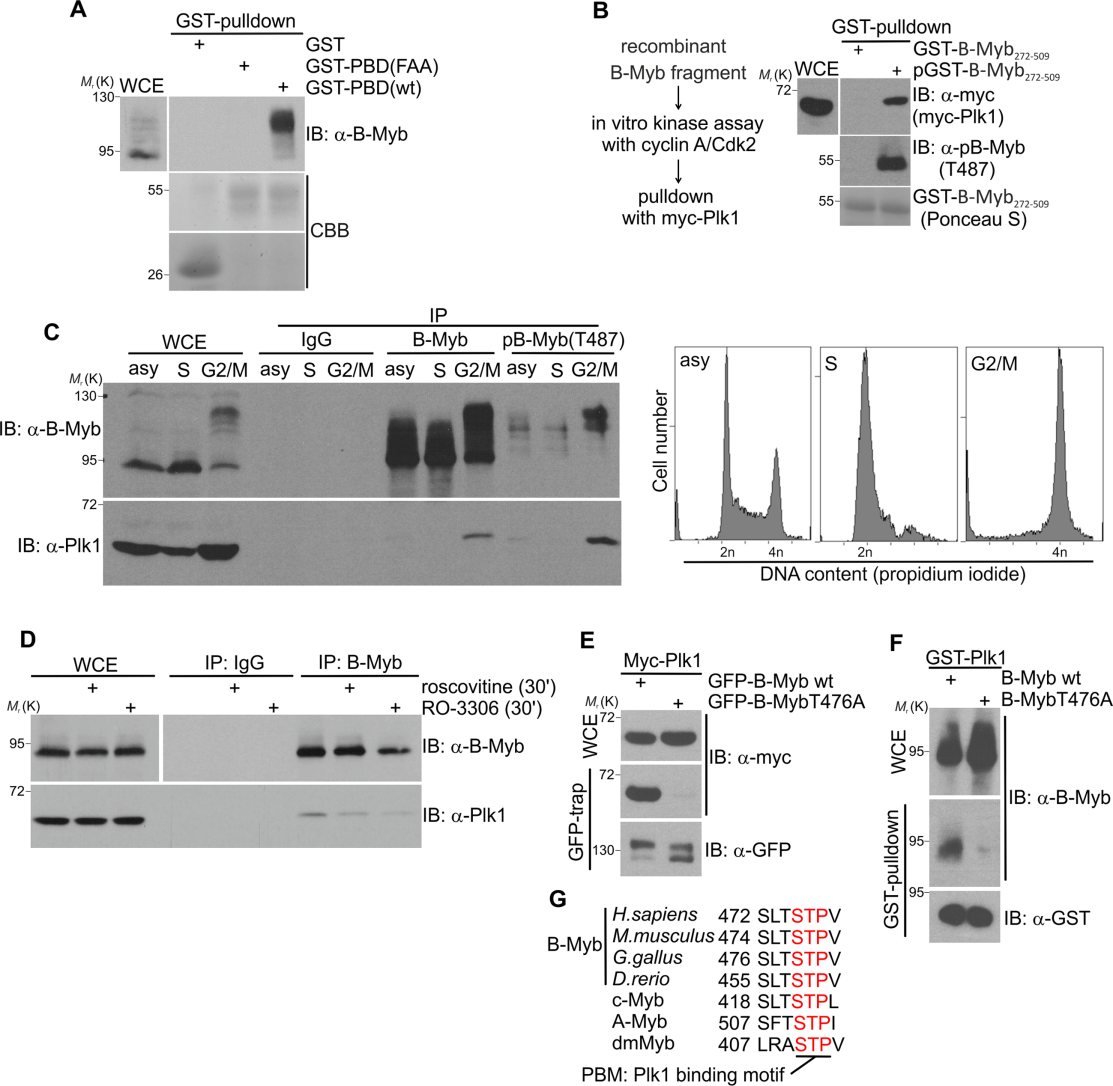
B-Myb interacts with Plk1 in the G2/M-phase through the conserved T476 phosphosite. (**A**) Whole cell lysates of Hek293 cells were incubated with glutathione-sepharose beads loaded with GST, GST-PBD(wt) or substrate-binding deficient GST-PBD(FAA) proteins. Bound proteins and WCE were analyzed by WB and Coomassie brilliant blue (CBB) staining as indicated. (**B**) Purified GST-B-Myb (aa 272–509) was loaded onto glutathione-sepharose beads, phosphorylated *in vitro* by cyclin A/Cdk2 and incubated with extracts from QT6 cells transiently expressing Myc-Plk1 (upper panel). Unphosphorylated GST-B-Myb was used as control. The phosphorylation of T487 was analyzed exemplarily to confirm the *in vitro* phosphorylation (middle panel). Ponceau S staining of bound GST-B-Myb (aa 272–509) is shown in the bottom panel. (**C**) HepG2 cells were enriched in S-phase by thymidine block and release for 1 h, or enriched in G2/M by treatment with RO-3306 for 18 h and release for 30 min or left untreated (asy). *Left:* Cell lysates were immunoprecipitated with pan-B-Myb (‘#53’), phospho-B-Myb(T487) or control IgG antibodies. Immunoprecipitates and WCE were analyzed by WB as indicated. *Right:* Cell cycle distribution was analyzed by flow cytometry. (**D**) Lysates of Hek293 cells pretreated with or without roscovitine or RO-3306 for 30 min were immunoprecipitated with B-Myb-specific (‘brian’) or control antibodies. Bound proteins and aliquots of the whole cell extracts (WCE) were analyzed by western blotting with antibodies against Plk1 and B-Myb. (**E**) Lysates prepared from QT6 cells transiently expressing Myc-Plk1 together with GFP-B-Myb(wt) or GFP-B-Myb(T476A) were immunoprecipitated with GFP-trap beads. Immunoprecipitates and WCE were analyzed by WB. (**F**) Lysates prepared from QT6 cells transiently expressing B-Myb(wt) or B-Myb(T476A) were incubated with glutathione-sepharose beads loaded with GST-Plk1. Bound proteins and WCE were analyzed by WB. (**G**) Amino acid sequence alignment surrounding T476 in B-Myb from different species, human c-Myb and A-Myb and dmMyb from *D. melanogaster*. The numbers refer to the first amino acids shown in each case.

Although cyclin-dependent kinases are the master regulators of the cell cycle they are supported by additional cell cycle dependent kinases. Polo-like kinase 1 (Plk1) is a member of the Polo family of mitotic kinases that regulates multiple stages of mitosis and cytokinesis and phosphorylates substrate proteins primed by the previous phosphorylation of Ser-Thr-Pro (STP) amino acid motifs. These motifs are recognized by the phospho-specific Polo box domain (PBD) when phosphorylated at the central Thr residue (47–49). Because B-Myb contains two STP motifs (see below) we wondered whether Plk1 binds to B-Myb following the Cdk-mediated phosphorylation of one or both of these motifs and thereby contributes to the hyper-phosphorylation of B-Myb in the G2/M-phase. In support of this idea we observed that the slow migrating, phosphorylated forms of endogenous B-Myb readily interacted *in vitro* with GST-PBD(wt), but not with the substrate-binding deficient GST-PBD(FAA) mutant (Figure 4A). By contrast, the faster migrating form of B-Myb failed to interact (Figure 4A). In a reverse binding experiment, Plk1 interacted with a bacterially expressed GST-B-Myb fragment which contains both STP motifs only after GST-B-Myb was phosphorylated *in vitro* by cyclin A/Cdk2 (Figure 4B). This confirms that phosphorylation of B-Myb is crucial for Plk1/B-Myb binding. Importantly, co-immunoprecipitation with Plk1- or B-Myb-specific antibodies and extracts from synchronized HepG2 or Hek293 cells revealed that Plk1 and B-Myb also interact when expressed endogenously and that the interaction occurs preferentially in the G2/M-phase (Figure 4C and Supplementary Figure S4A). Moreover, the phospho-T487 B-Myb antibodies co-immunoprecipitated endogenous Plk1 much more efficiently than two different pan-B-Myb antibodies, confirming the specific association of Plk1 with phosphorylated endogenous B-Myb (Figure 4C and Supplementary Figure S4B). Consistently, in the reverse co-immunoprecipitation experiment antibodies against Plk1 specifically precipitated the slower migrating forms of endogenous B-Myb phosphorylated at T487 (Supplementary Figure S4A). Collectively, these data provide strong evidence that B-Myb and Plk1 associate under physiological conditions in the G2/M-phase. Furthermore, the endogenous B-Myb/Plk1 association was diminished in cells which were pretreated with roscovitine or RO-3306 shortly before lysis, indicating that Cdk2- or Cdk1-mediated phosphorylation of B-Myb is essential for Plk1 binding (Figure 4D). Given that cyclin A was reported to be degraded early in mitosis (50,51) we wondered whether there was still cyclin A-associated kinase activity under conditions when B-Myb interacts with Plk1. Therefore, we enriched cells in early mitosis by STLC treatment and performed *in vitro* kinase assay with endogenous cyclin A immunoprecipitates in parallel to Plk1-coimmunoprecipitation. As expected, Plk1/B-Myb interaction was enhanced in STLC-treated cells (Supplementary Figure S4C). Importantly, the amounts of cyclin A/Cdk2 proteins (although reduced in STLC-treated cells) and the residual kinase activity were still sufficient to phosphorylate GST-B-Myb *in vitro*, as demonstrated exemplarily for T487 (Supplementary Figure S4D). This is also in line with the observations that Cdk2 activity rises up to the anaphase during the cell cycle and cyclin A levels are highest at the beginning of the prometaphase dropping gradually during prometaphase-anaphase transition (52,53). Thus we conclude that cyclin A/Cdk2 or -/Cdk1 might stimulate Plk1/B-Myb association *in vivo*.

Utilizing B-Myb deletion mutants we mapped the Plk1 docking site to aa 335–509 of B-Myb (Supplementary Figure S4E). This part of B-Myb contains both potential STP Plk1-binding motifs surrounding T444 and T476. Mutation analysis showed that only alanine substitution of T476 abolished Plk1 binding to B-Myb (Supplementary Figure S4F). Furthermore, full-length B-Myb(T476A) could neither co-immunoprecipitate Myc-Plk1 *in vivo* nor interact with GST-Plk1 *in vitro*, confirming the T476 site as the functional Plk1-binding motif (PBM) in B-Myb (Figure 4E and F). Notably, this binding motif shows strong sequence conservation across various organisms and other Myb proteins, highlighting its importance (Figure 4G).

### Cdk-mediated phosphorylation of B-Myb at T476 is stimulated by Pin1

**Figure 5.**
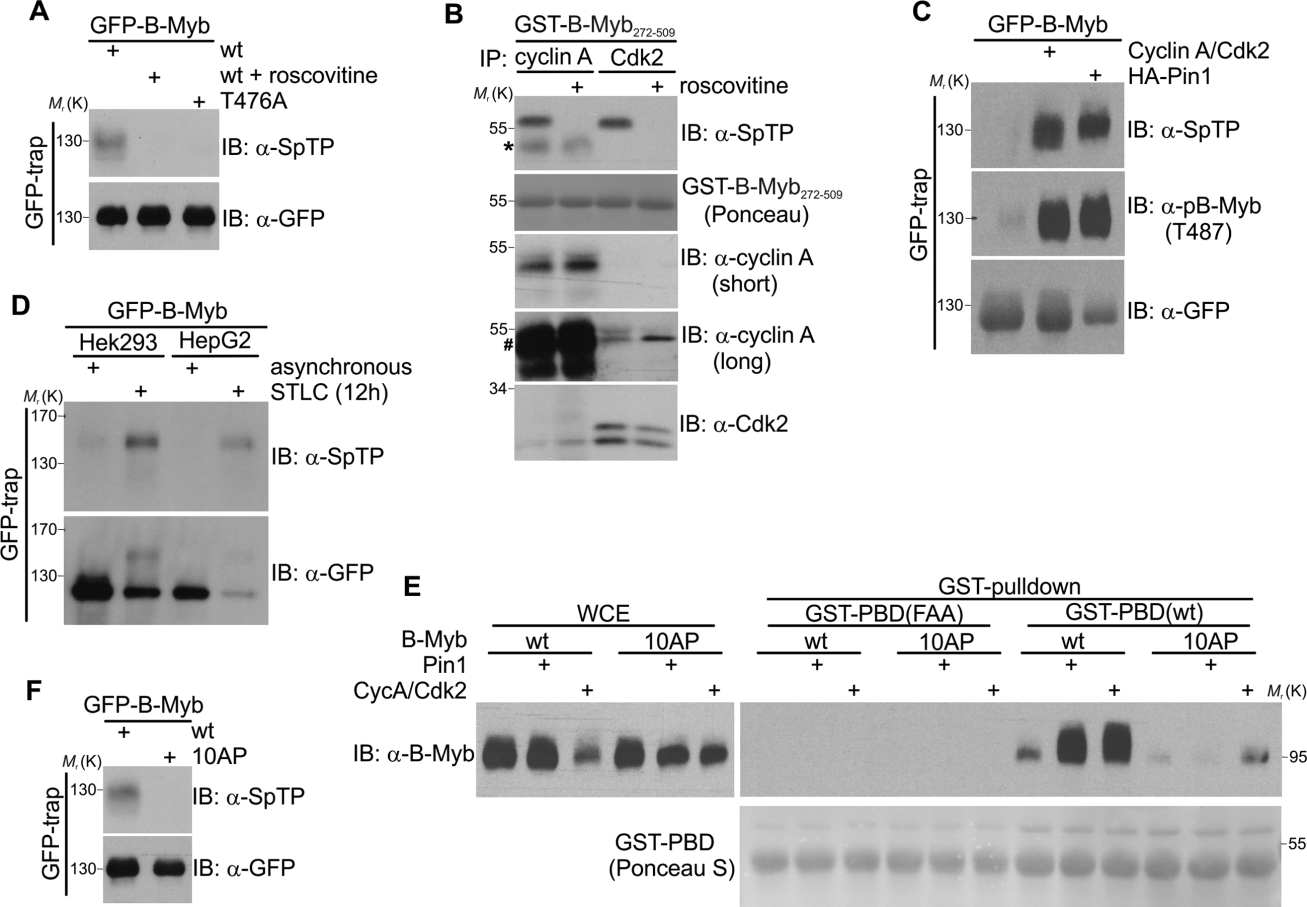
Phosphorylation of B-Myb at T476 is facilitated by Pin1. (**A**) GFP-B-Myb(wt) and GFP-B-Myb(T476A) were transiently expressed in QT6 cells, immunopurified with GFP-trap beads and analyzed by WB. Roscovitine was added to the cells 45 min prior to the lysis, as indicated. (**B**) *In vitro* kinase assay with recombinant GST-B-Myb (aa 272–509) fragment and endogenous cyclin A- or Cdk2-immunoprecipitates from Hek293 cells. Roscovitine was added to reaction mix as control of the assay specificity. The asterisk marks heavy chains from IgG used for cyclin A immunoprecipitation. Please note that cyclin A band overlaps with the IgG heavy chain, as indicated by #. (**C**) Lysates from QT6 cells transiently expressing GFP-B-Myb together with or without Pin1 or cyclin A/Cdk2 were immunoprecipitated with GFP-trap beads. Immunoprecipitates were analyzed by WB with the indicated antibodies. (**D**) Lysates from untreated or STLC-treated Hek293 and HepG2 cells stably expressing GFP-B-Myb were immunoprecipitated with GFP-trap beads. Immunoprecipitates were analyzed by WB with the indicated antibodies. (**E**) Lysates prepared from QT6 cells transiently expressing B-Myb(wt) or B-Myb(10AP) together with or without Pin1 or cyclin A/Cdk2 were incubated with glutathione-sepharose beads loaded with GST-PBD(wt) or GST-PBD(FAA). Bound proteins and WCE were analyzed by WB and Ponceau S staining. (**F**) GFP-B-Myb(wt) and GFP-B-Myb(10AP) were transiently expressed in QT6 cells, immunopurified with GFP-trap beads and analyzed by WB.

T476 is an as yet uncharacterized B-Myb phosphorylation site that has been detected in several high-throughput mass spectrometry studies. To analyze T476 phosphorylation we used antibodies recognizing the phosphorylated Plk1 binding motif (anti-SpTP). To be able to visualize SPT phosphorylation of B-Myb independently of other cellular proteins phosphorylated at such sites we employed a GFP-B-Myb fusion protein that could be efficiently immunopurified from cell extracts by binding to GFP-trap beads. Immunopurified GFP-B-Myb(wt), but neither GFP-B-Myb(wt) isolated from roscovitine-treated cells nor the T476A mutant, was readily recognized by the anti-SpTP antibody, confirming its specificity and validating it as readout for T476 phosphorylation when used with purified B-Myb (Figure 5A). Using this antibody and a recombinant GST-B-Myb fragment, we then demonstrated that T476 is phosphorylated by endogenous cyclin A- or Cdk2- immunoprecipitates *in vitro* which was fully prevented by roscovitine (Figure 5B). Also, ectopically co-expressed cyclin A/Cdk2 strongly stimulated phosphorylation of GFP-B-Myb on T476 *in vivo* (Figure 5C). Importantly, co-expressed Pin1 also increased T476 phosphorylation indicating that T476, like T487, belongs to the class of ‘late’ Cdk2 phosphosites in B-Myb (i.e. sites whose phosphorylation is facilitated by prior Pin1-mediated conformational changes of B-Myb) (Figure 5C). Indeed, stably expressed GFP-B-Myb isolated from mitotically enriched, STLC-treated Hek293 and HepG2 cells was strongly phosphorylated at the T476 site (Figure 5D). This prompted us to investigate whether Pin1 facilitates binding of Plk1 to B-Myb through stimulation of T476 phosphorylation. Thus, we performed pulldown experiments with GST-PBD and B-Myb expressed with or without Pin1 or cyclin A/Cdk2. As expected, cyclin A/Cdk2 and Pin1 both stimulated B-Myb binding to GST-PBD (Figure 5E). However, in case of the B-Myb(10AP) mutant which contains the intact T476 phosphosite but is defective in Pin1 binding Pin1 failed to stimulate the interaction with GST-PBD, consistent with the notion that prior Pin1-induced conformational changes in B-Myb facilitate T476 phosphorylation und subsequent Plk1 docking. Indeed, analysis of immunopurified GFP-B-Myb(10AP) demonstrated the lack of T476 phosphorylation in the 10AP mutant (Figure 5F). Again, this is in line with our observation that phosphosites like T487 or T476 are poorer substrates for cyclin A/Cdk2 in B-Myb(10AP) than in B-Myb(wt) (compare Figure 5F and Figure 2D). Collectively, our data suggest that Pin1-stimulated, Cdk-mediated phosphorylation of B-Myb at T476 is crucial for Plk1 binding to B-Myb.

### Plk1 mediates phosphorylation of the B-Myb transactivation domain

**Figure 6.**
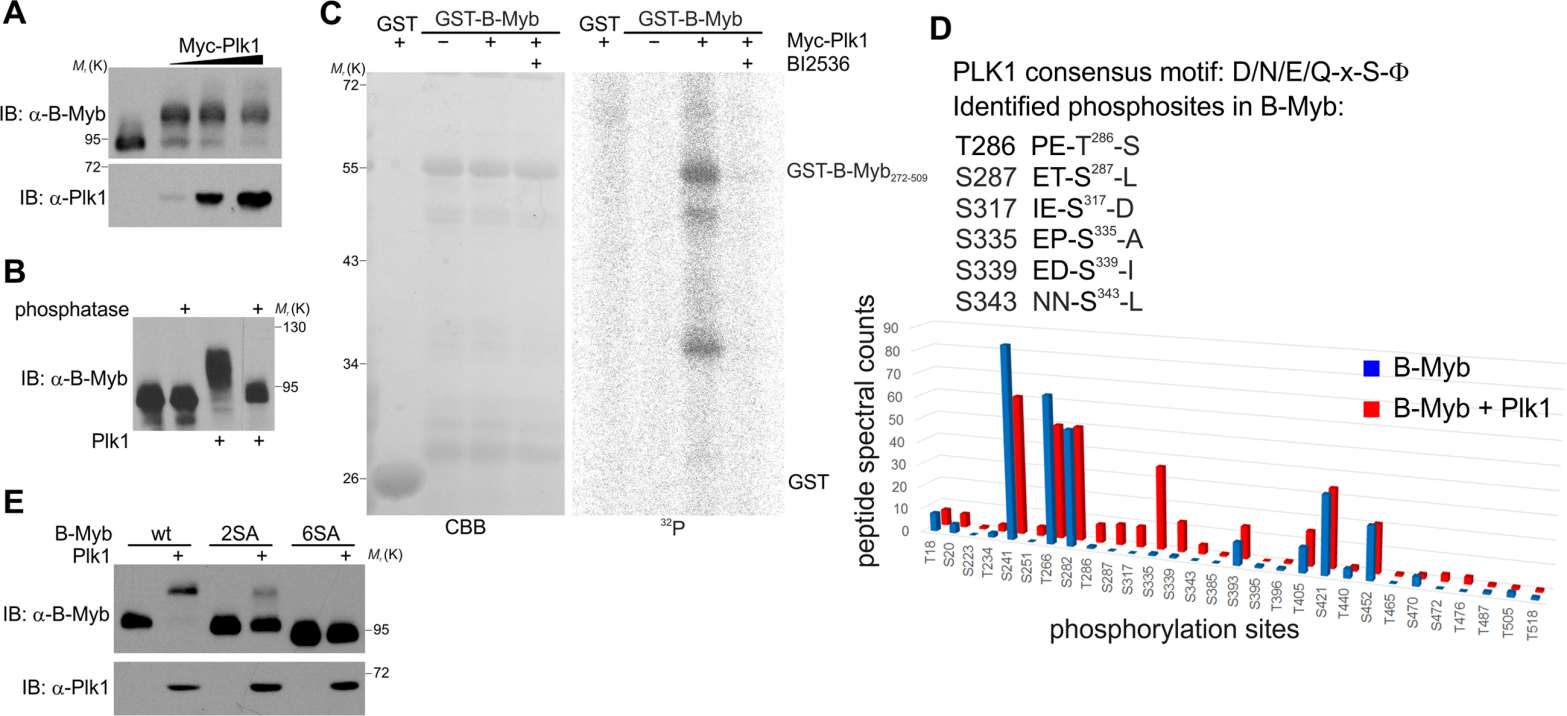
B-Myb is a direct target of Plk1 kinase. (**A**) Lysates of QT6 cells transiently expressing B-Myb together with increasing amounts Myc-Plk1 or without Myc-Plk1 were analyzed by WB. (**B**) Lysates of QT6 cells transiently expressing B-Myb together with or without Myc-Plk1 were treated with FastAP alkaline phosphatase or left untreated, followed by WB with B-Myb antibodies. (**C**) *In vitro* protein kinase assay. GST-B-Myb (aa 272–509) was phosphorylated *in vitro* with γ^32^P-ATP, using Myc-Plk1 immunopurified from transfected cells. Phosphorylated proteins were resolved by SDS-PAGE and visualized by autoradiography. A CBB-stained gel of the GST-proteins is shown on the left. GST served as substrate control. Immunoprecipitates from untransfected cells and a kinase reaction with the Plk1-specific inhibitor BI2536 served as additional controls. (**D**) *Left*: Plk1 consensus motif and identified B-Myb phosphosites enriched upon Plk1 expression are shown. The numbers refer to amino acids in human B-Myb. *Right*: Quantitative analysis of identified phosphosites. Phosphopeptide spectral counts resulting from quadruplicate analyses are shown. (**E**) Lysates from QT6 cells transiently expressing B-Myb(wt), B-Myb(2SA) or B-Myb(6SA) together with or without Myc-Plk1 were analyzed by WB.

Co-expression of B-Myb and Plk1 causes a strong phosphatase-sensitive electrophoretic mobility shift of B-Myb indicating that Plk1 induces phosphorylation of B-Myb (Figure 6A and B). To demonstrate that B-Myb is a direct Plk1 target we performed *in vitro* kinase assays with a bacterially expressed GST-B-Myb fragment and immunopurified Plk1. B-Myb was readily phosphorylated by Plk1, but not in the presence of Plk1 inhibitor BI2536 (Figure 6C). Moreover, bacterially expressed GST-Plk1 phosphorylated B-Myb in the absence of other eukaryotic proteins, indicating that Plk1 phosphorylates B-Myb directly (Supplementary Figure S5A).

Results from *in vitro* kinase assays suggested that Plk1 phosphorylates the central region of B-Myb containing the transcriptional activation domain (TAD) (Figure 6C). B-Myb has 14 potential phosphosites matching the Plk1-consensus site (D/E/N/Q-x-S/T-Φ) with 10 of them located in TAD region (54). We employed mass spectrometry of immunopurified GFP-B-Myb expressed with or without Plk1 to eventually identify six Plk1-stimulated phosphosites, all of which are located in the TAD and four of them follow the Plk1-consensus sequence (Figure 6D and Supplementary Figure S5B-C). Importantly, alanine substitution of two main (S335/S339) or all six residues strongly impaired or totally abolished the Plk1-induced mobility shift of B-Myb (Figure 6E). We concluded that Plk1 directly phosphorylates at least six residues located in the B-Myb TAD.

### Functional analysis of Cdk2- and Plk1-dependent phosphorylation of B-Myb

Previous studies on the phosphorylation-dependent activity of B-Myb so far mainly focused on Cdk-mediated phosphorylation and assessed the activity of phospho-deficient B-Myb mutants through artificial reporter assays that may not exactly reflect the physiological situation (17,21,22). Furthermore, the Plk1-dependent aspect of B-Myb phosphorylation has not been explored yet. Thus, we next asked how phosphorylation of B-Myb by Plk1 modulates the function of B-Myb as a cell cycle regulator.

**Figure 7.**
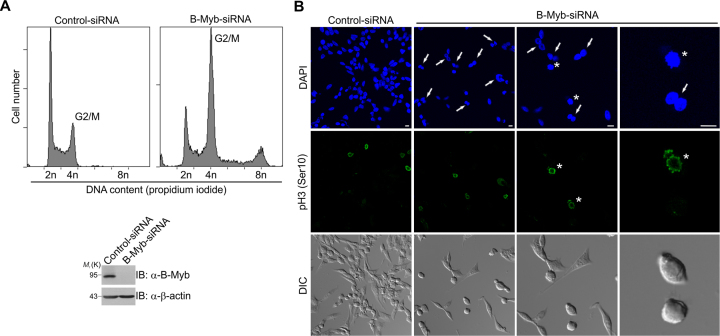
G2/M cell cycle arrest and formation of binucleated cells upon B-Myb knockdown. (**A**) Hek293 cells were transfected with control or B-Myb-specific siRNAs, incubated for 48 h and analyzed by WB (*left*) or flow cytometry (*right*). (**B**) Hek293 cells were transfected as in A, stained with DAPI for DNA or with phospho-histone H3(S10) antibody for mitotic cells and analyzed by confocal microscopy. Arrows and stars indicate binucleated and mitotic cells, respectively. DIC, Differential interference contrast; scale bars, 10 μm.

Generally, down-regulation of B-Myb leads to various mitotic abnormalities caused by severe mitotic spindle and centrosome defects and resulting in mitotic arrest, failure in cytokinesis and polyploidy (19,55). We confirmed these results in Hek293 cells by an RNAi approach and flow cytometric analysis of cell cycle progression. Hek293 cells transfected with B-Myb specific siRNA showed a strong accumulation of G2/M phase cells and obvious formation of multiploid cells with 8N DNA content (Figure 7A). Microscopic analysis revealed the formation of binucleated cells induced by B-Myb depletion (Figure 7B). Using an antibody against phospho-histone H3(S10) to specifically stain mitotic cells, we further observed that binucleated cells were no longer pH3(S10)-positive, indicating that following G2/M arrest/delay the B-Myb-depleted cells undergo mitosis without cytokinesis, resulting in G1-like tetraploid cells (Figure 7B, right panels). Similar results were obtained using an another human cell line, PC3, as well as using an independent B-Myb-specific siRNA (Supplementary Figure S6), suggesting that G2/M arrest and binucleated phenotype are general cell responses to loss of B-Myb.

**Figure 8.**
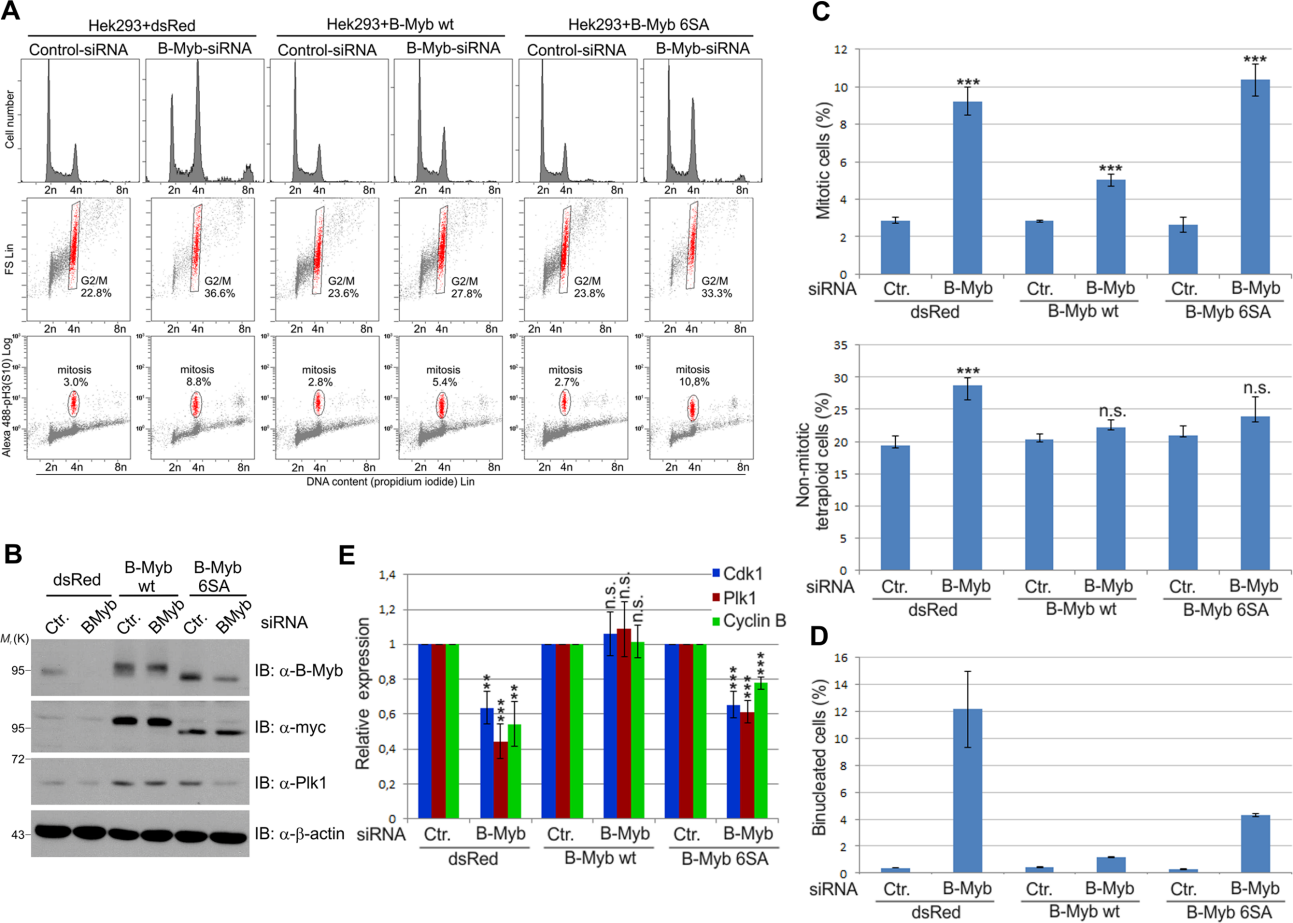
Plk1-dependent phosphorylation of B-Myb is essential for the expression of pro-mitotic B-Myb target genes. (**A** and **B**) Hek293 cells stably expressing dsRed, Myc-B-Myb(wt) or Myc-B-Myb(6SA) were transfected with control or B-Myb-specific siRNAs. After 48 h cells were analyzed by WB (B) and flow cytometry (A) with phospho-histone H3(S10) antibody and propidium iodide. (**C**) The cells characterized in panel A were used to determine the percentage of mitotic (pH3(S10)-positive) (*top*) and non-mitotic, tetraploid (4n) cells (*bottom*). Values reflect the mean ± standard deviation of three independent experiments. (**D**) The cells characterized in panel A were stained with DAPI for DNA and analyzed by confocal microscopy to determine the percentage of binucleated cells. Values reflect the mean ± standard deviation of two independent experiments (1497–1935 cells scored per condition). (**E**) Analysis of B-Myb target gene expression of the cells characterized in panel A. Total RNA was extracted 48 h after siRNA transfection and analyzed by RT-PCR. mRNA expression levels were first normalized to *TBP* as a reference gene. Subsequently, values from B-Myb siRNA-transfected cells were normalized to control-siRNA transfected cells. Values are mean with standard deviation from three independent experiments. Statistical significance was determined by unpaired *t*-test. **P <0.01; ***P <0.001; n.s., not significant.

Next, we employed lentiviral transduction to establish Hek293 cells expressing myc-tagged, siRNA-resistant wt or mutant forms of B-Myb, enabling us to perform RNAi-based rescue assays. We first focused on the B-Myb(6SA) mutant lacking 6 Plk1-dependent phosphosites. As control, cells were transduced with the parental lentivirus expressing dsRed. Transduced cells were then transfected with control or B-Myb-specific siRNAs and analyzed by western blotting and flow cytometry. Endogenous B-Myb was efficiently silenced in Hek293-dsRed cells, while exogenous myc-tagged B-Myb was not affected (Figure 8B). As expected, B-Myb knockdown in control dsRed cells strongly perturbed normal cell cycle progression which was restored in B-Myb(wt) expressing cells. Interestingly, B-Myb(6SA) only partially rescued loss of endogenous B-Myb, as evidenced by the increased amounts of G2/M phase cells compared to cells rescued by B-Myb(wt) (Figure 8A, upper panels). This prompted us to perform further cell cycle analyses using an antibody against pH3(S10) to quantitate mitotic cells simultaneously to DNA content. This enabled us to further dissect the tetraploid G2/M population into pH3(S10)-positive (i.e.mitotic cells) and pH3(S10)-negative cells (i.e. non-mitotic G2 cells that have not yet entered into mitosis or bi-nucleated G1-like cells that have passed mitosis without undergoing cytokinesis). B-Myb knockdown in control dsRed cells increased the number of mitotic cells, which was significantly reduced in B-Myb(wt) expressing cells (Figure 8A, bottom panels and Figure 8C, top diagram). Interestingly, the mitotic index of B-Myb(6SA) expressing cells was comparable to that of dsRed cells, indicating that B-Myb(6SA) did not rescue mitotic defects. We then calculated the percentage of non-mitotic tetraploid cells, i.e. the difference between total and mitotic tetraploid populations (Figure 8A, middle and bottom panels and Figure 8C). Importantly, this showed that non-mitotic tetraploid cells accumulated after B-Myb knockdown in dsRed cells, but not in B-Myb(wt) or B-Myb(6SA) cells (Figure 8C, bottom diagram). As pointed out above, accumulation of such cells might be due to G2-arrest before mitotic entry or to failure in cytokinesis, resulting in the binucleated phenotype. Thus, we determined the percentage of binucleated cells by microscopic analysis. Interestingly, this showed that dsRed cells displayed a strong increase of binucleated cells, which was fully prevented by B-Myb(wt) and only slightly increased in B-Myb(6SA) expressing cells (Figure 8D). Assuming defective cytokinesis as cause of binucleation we conclude that dsRed cells suffer from both mitotic and cytokinetic defects after B-Myb knockdown, while the B-Myb(6SA) mutant rescues cytokinesis, but not the mitotic defects. Since B-Myb was implicated to have transcription-dependent and -independent functions in mitosis, we next examined the expression of B-Myb target genes via quantitative RT-PCR. We found that B-Myb knockdown in dsRed cells significantly reduced the expression of the key B-Myb target genes *PLK1, CCNB1* and *CDK1*, which was fully rescued by B-Myb(wt) but not by B-Myb(6SA) (Figure 8E). Collectively, our data indicate that Plk1-dependent phosphorylation of B-Myb is crucial to engage B-Myb for transcriptional activation of pro-mitotic genes, while being dispensable for the cytokinesis-related B-Myb function.

**Figure 9.**
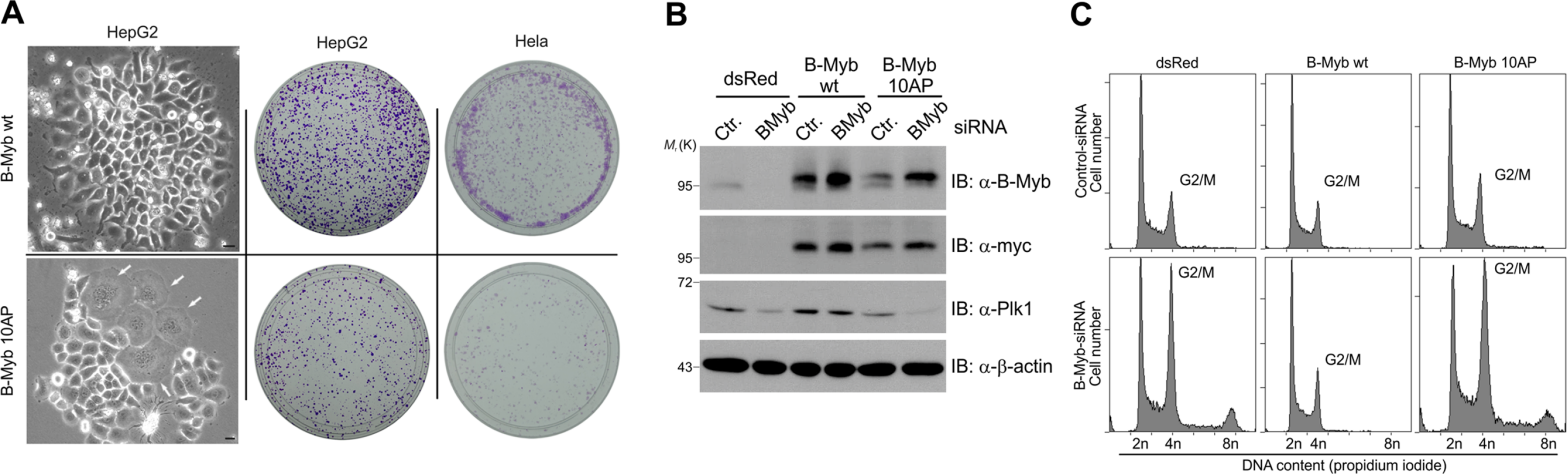
Analysis of B-Myb(10AP) expressing cells with or without knockdown of endogenous B-Myb. (**A**) HepG2 or Hela cells were infected with a lentivirus coding for Myc-B-Myb(wt) or Myc-B-Myb(10AP) for 48 h, followed by puromycin selection for additional 48 h. 10^4^ of the resulting cells were replated and cultured for 7 days. *Left:* Phase contrast pictures of colonies of Myc-B-Myb(wt) and Myc-B-Myb(10AP) expressing HepG2 cells. Arrows indicate multinucleated cells. *Right:* Cell colonies were fixed with formaldehyde and stained with crystal violet. (**B and C**) Hek293 cells were infected with lentivirus coding for dsRed, Myc-B-Myb(wt) or Myc-B-Myb(10AP) for 24 h, selected with puromycin for additional 24 h, and transfected with B-Myb-specific or control siRNAs. After 48 h, cells were analyzed by WB (B) or flow cytometry (C).

Since our work has implicated the Cdk-dependent phosphorylation of B-Myb in the Pin1-facilitated recruitment of Plk1 we wondered whether the Cdk-phosphosites serve additional functions, especially in light of the large number of these sites. We addressed the functional relevance of Cdk-dependent B-Myb phosphorylation by establishing Hek293 cells stably expressing the B-Myb(10AP) mutant lacking 10 Cdk phosphosites. A mouse homologue of this mutant was previously tested in reporter gene experiments and reported to be strongly impaired in its transactivation function (21). Unexpectedly, during initial efforts to establish stable cell lines we noted strong growth inhibition of cells expressing the B-Myb(10AP) mutant, indicating that the B-Myb(10AP) protein interferes with endogenous B-Myb in a dominant negative manner. This effect was observed in different cell lines, like HepG2, Hela or Hek293 and was accompanied by formation of multinucleated cells (Figure 9A). A similar dominant negative effect in reporter gene experiments was also reported for a mouse B-Myb mutant lacking 15 Cdk2 phosphosites (22). To minimize long-term effects of B-Myb(10AP) expression, we performed RNAi-mediated rescue assays immediately after lentiviral transduction and analyzed the cells by western blotting and flow cytometry (Figure 9B and C). Again, B-Myb knockdown in dsRed cells caused G2/M cell cycle arrest which was completely prevented in B-Myb(wt) expressing cells (Figure 9C). Interestingly, B-Myb(10AP) mutant failed to rescue loss of endogenous B-Myb, fully phenocopying the cell cycle defects in dsRed cells. Consistently, B-Myb(10AP) failed to rescue the reduced expression of the B-Myb target gene *PLK1* (Figure 9B). Thus, we concluded that Cdk-dependent phosphorylation of B-Myb is not only required to recruit Plk1 but mediates further essential functions of B-Myb in the cell cycle progression.

### DREAM/MMB complex perturbations in cancer

**Figure 10.**
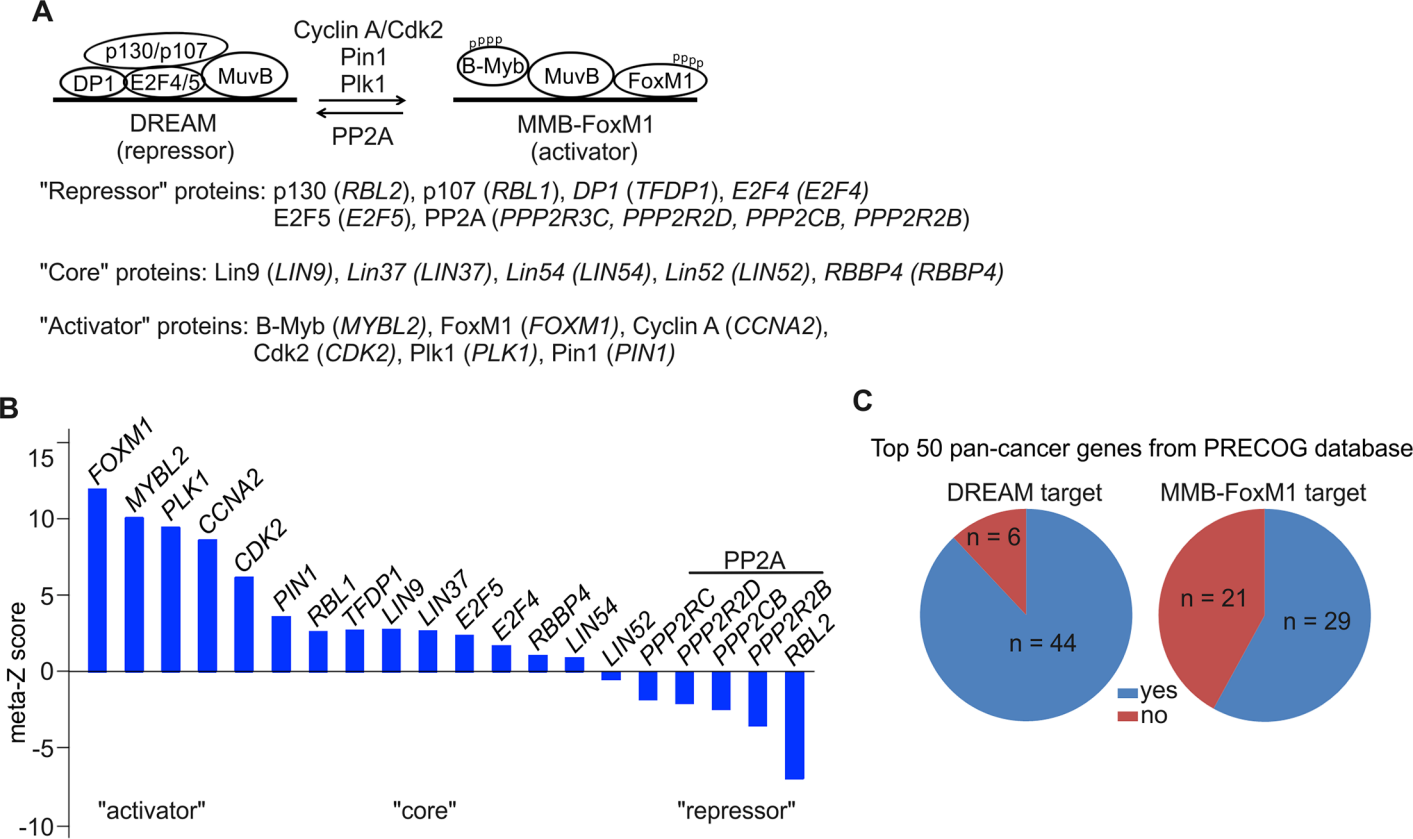
DREAM/MMB complex perturbations in cancer. (**A**) Schematic view of the balance between DREAM repressor and MMB-FoxM1 activator complexes is shown. B-Myb is shown displaced from the DNA to indicate that there is still no clear evidence that its DNA binding activity contributes to the binding of the MMB complex, as discussed recently (56). The proposed functions of indicated proteins were derived from literature and this study. The proteins (and the corresponding genes) are classified below as repressor, core and activator proteins of these complexes. Please note that exact functions of some genes are still poorly understood. (**B**) Meta-*Z* scores of DREAM/MMB-FoxM1-relevant genes specified in A, showing specific clustering of functionally related genes. (**C**) Top 50 pan-cancer genes as identified by PRECOG analysis were classified as targets of regulation by DREAM or MMB-FoxM1 complexes.

As indicated in the introduction, the balance between the repressive DREAM complex and the activating Myb-MuvB (MMB) complex is frequently perturbed in cancer (1). Overexpression of several proteins that play crucial roles as components or regulators of the MMB complex, including B-Myb (56–59), FoxM1 (60,61) and Plk1 (62), have previously already been shown to correlate with aggressive tumor phenotype and poor prognosis for patients. To further substantiate the clinical relevance of the B-Myb/FoxM1/Cdk/Plk1 axis we applied PRECOG, a pan-cancer resource of expression signatures that correlates cancer gene expression and clinical prognosis data through calculation of meta-z-scores (29). To obtain a DREAM/MMB prognostic pattern we assessed global meta-z scores across different cancers for genes related to the activity of the DREAM/MMB pathway. These genes (and their encoded proteins) were subdivided into ‘activator’, ‘core’ and ‘repressor’ according to their proposed function in the context of the DREAM/MMB complexes being either parts of the respective complexes, e.g. MYBL2/FoxM1 or E2F4/E2F5 or modulating the activity of the single subunits, e.g. PLK1 or PP2A (Figure 10A) (1,56,63,64) (and results from this study). Interestingly, we found specific clustering of functionally related genes in terms of survival prognosis (Figure 10B). In fact, increased expression of activator genes correlated with adverse clinical outcome, while expression of most repressor genes was associated with a favorable prognosis. Additionally, we analyzed top 50 pan-cancer genes from PRECOG associated with poor survival and further classified them as DREAM/MMB targets (Figure 10C and Supplementary Figure S7) (30). Remarkably, 44 out of 50 genes (including *MYBL2, FOXM1, CCNA2* and *PLK1*) were found to be targets of DREAM-mediated repression, while 29 of them were also targets of MMB activation (Figure 10C). These data accord with the prevailing notion that DREAM/MMB complex perturbations and the associated gene expression changes are highly relevant for tumor development and may help to delineate cancers with poor prognosis.

## DISCUSSION

**Figure 11.**
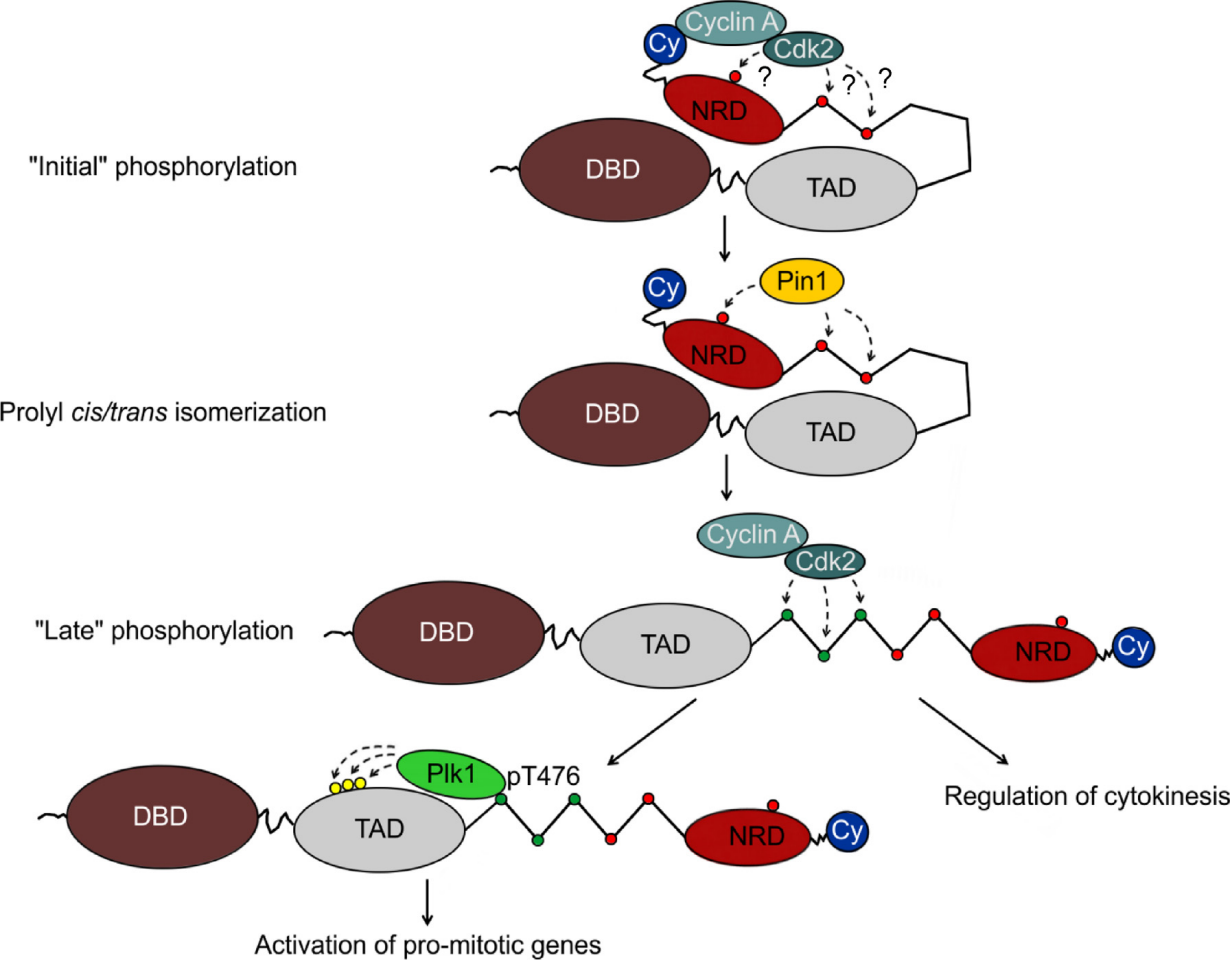
Hypothetical model of cell cycle-dependent B-Myb activation. B-Myb is depicted with its three major domains including the N-terminal DNA-binding domain (DBD), the transactivation domain (TAD) and the C-terminal negative regulatory domain (NRD). Unphosphorylated B-Myb is assumed to adopt a ‘closed’ kinase-restricted conformation, which is allows initial, presumably Pin1-independent Cdk-induced phosphorylation at one or several sites, whose positions in B-Myb are presently not known (marked by ‘?’). Subsequent Pin1 binding to these sites and Pin1-induced conformational changes facilitate further Cdk-dependent phosphorylation (including phosphorylation at T476). Binding of Plk1 then allows phosphorylation at sites in the transactivation domain of B-Myb to finally stimulate transcription of late cell cycle genes, while Cdk-phosphorylated B-Myb functions more broadly in mitotic regulation and cytokinesis. The C-terminal Cy motif in B-Myb might be particularly important under low, but redundant under high cyclin A-dependent kinase levels.

We have uncovered a complex, stepwise mechanism of cell cycle-dependent B-Myb phosphorylation which is mediated by Cyclin-dependent kinase and Polo-like kinase 1 and orchestrated by the prolyl *cis/trans* isomerase Pin1. Our data show that Pin1 binds to B-Myb in a Cdk-phosphorylation-dependent manner and induces conformational changes to increase the Cdk-dependent phosphorylation at specific sites, as demonstrated by the T476 and T487 phosphosites. That this increase is mediated by the interaction of Pin1 with B-Myb itself (and not effects of Pin1 on the activity of other proteins) was ascertained by the observation that the Pin1 binding-deficient B-Myb(10AP) mutant failed to show a similar increase (Figure 2E); furthermore, the overall cyclin A-dependent kinase activity was not increased by Pin1 (Figure 2D). As proposed by the hypothetical model depicted in Figure 11, we therefore assume that unphosphorylated B-Myb adopts a kinase-restricted (or ‘closed’) conformation, which only permits initial (or ‘early’) phosphosites to be phosphorylated by cyclin A/cdk in a Pin1-independent manner, allowing then binding of Pin1 and Pin1-mediated conformational changes in B-Myb. The identitiy of the ‘early’ site(s) is currently not known and their phosphorylation may be facilitated by the Cy motif that we have identified. The Pin1 induced conformational changes in turn enable subsequent Cdk-mediated phosphorylation on ‘late’ phosphosites including T476, which then creates a docking site for Plk1 and triggers further phosphorylation by Plk1, committing B-Myb to activate pro-mitotic genes. Importantly, the Cdk2-dependent phosphorylations serve not only to recruit Plk1 but also enable B-Myb to function more broadly in mitotic regulation and cytokinesis. Interestingly, the identified regulatory elements (Cy and PBM motifs) show high evolutionary conservation across the Myb family. Moreover, the transcriptional activity of other Myb family members can be stimulated by cyclin A/Cdk2 or Pin1 (27,65). Thus, the regulatory principles uncovered here might also apply to other Myb family members. In this context it is interesting that C-terminal truncations of B-Myb have not been shown to be tumorigenic so far, as opposed to c-Myb and A-Myb. One might speculate that the strong c-Myb and A-Myb transcriptional activation domains do not require phosphorylation by Plk1 for transcriptional activation. In contrast, the weaker transactivation domain of B-Myb appears to require phosphorylation by Pkl1, which presumably is not possible in C-terminally truncated B-Myb. However, it is also possible that tumors harboring C-terminal truncations of B-Myb have not been found because this would disrupt the B-Myb/Lin9 interaction and its association with the MuvB complex.

Several aspects of the proposed model are not yet fully understood: It remains to be addressed how the proposed ‘closed’ conformation in B-Myb is achieved on the molecular level. Previous work has revealed that the C-terminal part of B-Myb functions as a negative regulatory domain, which might be involved in establishing the kinase-restricted conformation of B-Myb. Interestingly, an intramolecular interaction between the DNA-binding domain (DBD) and the so-called EVES motif which is embedded in the negative regulatory domain (NRD) was reported for c-Myb (66), suggesting that similar mechanism might also serve to hold B-Myb in a ‘closed’ conformation. A further question concerns the identity of the Cdk-phosphosites whose phosphorylation is independent of Pin1-induced conformational changes during the initial phosphorylation of B-Myb. Addressing this question is currently hampered by the lack of phosphorylation-specific antibodies against the majority of B-Myb phosphorylation sites. Additionally, it is currently unclear whether Cdk2 is solely responsible for B-Myb phosphorylation during the unperturbed cell cycle. Studies in mice have revealed that Cdk2 is dispensable for mitotic cell division, which contrasts the lethal phenotype upon loss of B-Myb and indicates that Cdk2’s function with regard to phosphorylation of B-Myb can be compensated by an another kinase, presumably by Cdk1, which like B-Myb, is also essential for cell proliferation (67,68). Unfortunately, it still remains experimentally challenging to clearly dissect Cdk1/Cdk2 activities in mammalian cells (40). Thus, we cannot exclude that both cyclin A-associated kinases contribute to phosphorylation of B-Myb. However, the fact that Cdk2 is a preferred binding partner for cyclin A, while Cdk1 only binds after Cdk2 saturation, predetermines the order of Cdk1/Cdk2 kinase activity (69,70). Nevertheless, our proposed stepwise phosphorylation mechanism can be easily adapted to both kinases, acting either alternatingly or simultaneously.

In a broader sense, our data also suggest that distinct phosphorylation patterns fine-tune B-Myb activity towards different functions or environmental demands. Previously, we found that B-Myb is partially dephosphorylated upon DNA damage, which coincides with transcriptional shutdown during recovery time (23). Such dephosphorylation can be a consequence of DNA damage-induced Cdk2 inhibition and prevent Pin1 recruitment to B-Myb, thereby abolishing its activation and ensuring a delay of the cell cycle progression. Interestingly, G2 checkpoint recovery requires residual Cdk activity to maintain a critical level of G2/M gene transcription (71). FoxM1 was identified as one of the Cdk targets that partially contributes to the maintenance of recovery competence, suggesting that B-Myb might be the missing factor to fully restore G2/M gene expression after DNA damage (71,72). In this setting, the Cy motif of B-Myb could be particularly important for cyclin A/Cdk2 or cyclin A/Cdk1 recruitment to B-Myb under low levels of cyclin A dependent kinases. As transcription of *CCNA2 (*cyclin A) and *CDK1* genes is stimulated by B-Myb and FoxM1, we expect a positive feedback loop increasing Cdk activity to a level that is necessary to inactivate the G2 checkpoint and restart the cell cycle (73). Notably, Plk1 was also implicated in the G2 checkpoint recovery, suggesting a similar B-Myb-dependent feedback mechanism (74–76). Apart from being involved in G2 checkpoint recovery Plk1 plays a key role as a regulator of multiple targets that execute various aspects of mitosis and cytokinesis (48,49). The positive B-Myb dependent feedback loop for Plk1 expression may therefore also be relevant for the progression through the unperturbed cell cycle as it may ensure the irreversibility of the G2/M transition.

We have shown that the phenotypes induced by expression of Plk1- and Cdk-phospho-deficient B-Myb mutants are strikingly different indicating that the Cdk-induced phosphorylations serve additional, currently unknown functions beyond mediating the recruitment of Plk1. We have found that Plk1-phospho-deficient B-Myb was defective in mitotic gene expression but was still able to maintain the execution of cytokinesis, unlike the Cdk-phospho-deficient B-Myb. The identified Plk1-dependent phosphosites are located in the TAD region of B-Myb, suggesting that they enhance the transactivation potential of B-Myb which might explain their requirement for optimal mitotic gene expression. The effect of the 6SA mutation on B-Myb target gene expression was only gradual, leading to decreased levels of these targets. If the threshold levels of crucial B-Myb targets (for example of Plk1) for the execution of different stages of mitosis were different this might explain why the 6SA mutant retards mitosis but has only little effect on cytokinesis. Alternatively, it is possible that the more extensive phenotype induced by expression of Cdk-phospho-deficient B-Myb 10AP mutant, which includes defective cytokinesis, is due to additional, Cdk phosphorylation-dependent functions of B-Myb that may not be related to the transcriptional activity of B-Myb. For example, it has been proposed that B-Myb carries out non-transcriptional roles during mitosis and cytokinesis. These authors demonstrated that a B-Myb deletion mutant (lacking amino acids 509–628 of murine B-Myb) was still able to transactivate B-Myb target genes but was unable to form a so-called Myb-Clafi complex and could not rescue the major cell cycle defects upon loss of B-Myb, leading to formation of binucleated cells (19). These results are reverse-complementary to our observations with the Plk1-phospho-deficient B-Myb(6SA) mutant which in turn was unable to stimulate gene expression (probably leading to mitotic delay) but could still prevent the failure in cytokinesis. Thus, it is conceivable that the additional as yet uncharacterized, Cdk phosphorylation-dependent functions of B-Myb are related to non-transcriptional roles of B-Myb in mitosis or cytokinesis, such as the proposed formation of the Myb-Clafi complex (19). In any case, our work suggests that the execution of different functions of B-Myb might be coordinated through different phosphorylation patterns, which contrasts the current ‘all or nothing’ model of B-Myb activation by phosphorylation.

Finally, increased B-Myb expression has been reported for a variety of tumor types and is thought to reflect an imbalance of the switch between DREAM and MMB-FoxM1 complexes (1,56,57). PRECOG analysis not only confirms the notion of high B-Myb expression as a predictor of poor prognosis for tumor patients but also shows that the expression of several factors involved in B-Myb activation is associated with an adverse fate. Similarly, PRECOG strongly implicates the expression of genes regulated by the DREAM and MMB-FoxM1 complexes in tumorigenesis, emphasizing the prevailing view of a pro-tumorigenic role of B-Myb and the MMB complex. Interestingly, we also noted that our sequential phosphorylation mechanism has remarkable similarities to regulation of FoxM1 activity. FoxM1 is an autorepressed transcription factor, which is activated by initial cyclin A/Cdk and subsequent Plk1 phosphorylation to stimulate late cell cycle gene expression (77–79). Very recently, Pin1 was reported to stimulate FoxM1 activity in metastatic melanomas and chemo-resistant cervical cell lines (80,81). Thus, we propose that the Cdk/Pin1/Plk1 axis is a common feature of the activation of mitotic transcription factors, which may also be helpful to identify cancers with poor survival prognosis.

## Supplementary Material

Supplementary Data
