# Supplementary material for: Activation of the oncogenic transcription factor B-Myb via multisite phosphorylation and prolyl cis/trans isomerization
Source: Nucleic Acids Res. 2018 Oct 13;47(1):103–21. doi: 10.1093/nar/gky935 (PMC6326806; doi:10.1093/nar/gky935)
Supplement: Supplementary Data [file gky935_supplemental_files.pdf]

Activation of the oncogenic transcription factor B-Myb via multisite phosphorylation and prolyl *cis*/*trans* isomerization

Supplementary Information

Eugen Werwein[1], Hannah Cibis[1], Daniel Hess[2] and Karl-Heinz Klempnauer[1]

[1]Institute for Biochemistry Westfälische-Wilhelms-Universität, D-48149 Münster, Germany

[2]Friedrich Miescher Institute for Biomedical Research, Maulbeerstr. 66, CH-4058 Basel, Switzerland

Fig.S1

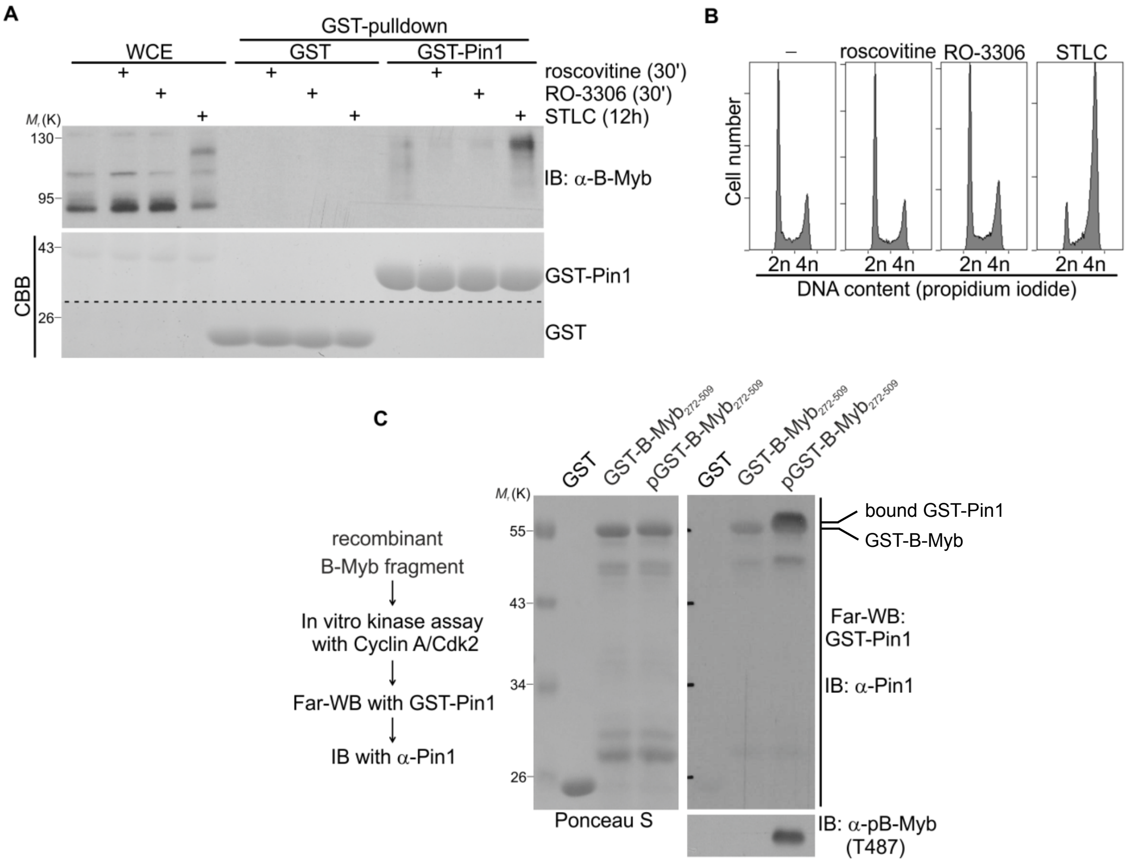

Fig S1. Pin1 interacts with B-Myb in a phosphorylation-dependent manner

A) Lysates of HepG2 cells pretreated with or without roscovitine or RO-3306 for 30 min or treated with STLC for 12h were incubated with glutathione-sepharose beads loaded with GST or GST-Pin1. Bound proteins and aliquots of the whole cell extracts (WCE) were analyzed by western blotting with antibodies against B-Myb. Loading of the beads with GST-proteins was confirmed by Coomassie Brilliant Blue (CBB) staining of the blot.

B) Cell cycle profiles of Hek293 cells treated as described in Fig **1C**.

C) GST-B-Myb (aa 272-509) was phosphorylated in vitro by cyclin A/Cdk2 as confirmed by anti-phospho-B-Myb(T487) blot (bottom) and then subjected to Far-WB analysis with GST-Pin1, followed by probing with Pin1-specific antibodies. GST and unphosphorylated GST-B-Myb were used as controls.

Fig.S2

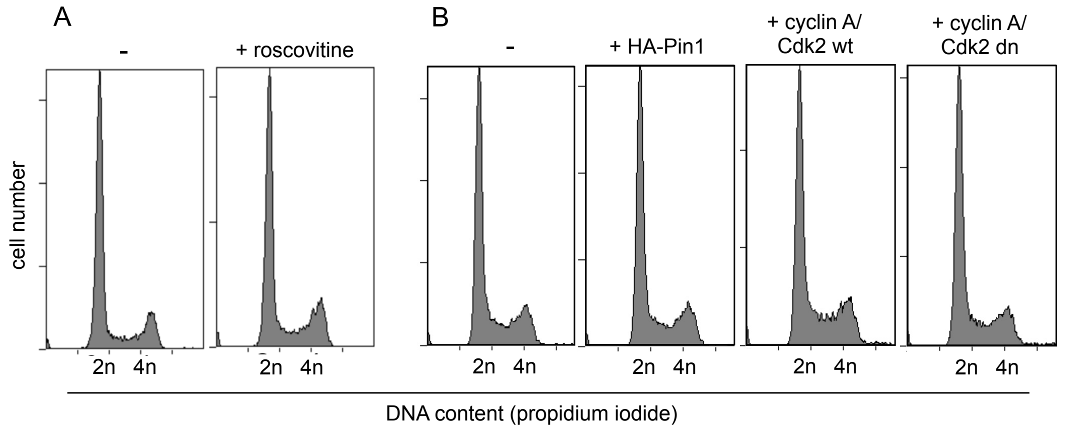

Fig S2. Cell cycle profiles of transfected or untransfected Hela cells

Hela cells were treated with roscovitine as in Fig.2C (A) or transfected the same concentrations of the indicated DNAs as in Fig.2C (B), using Lipofectamine as transfection reagent. Under these conditions the majority of the cells took up the DNA. The cells were stained with propidium iodide and analyzed by flow cytometry.

Fig.S3

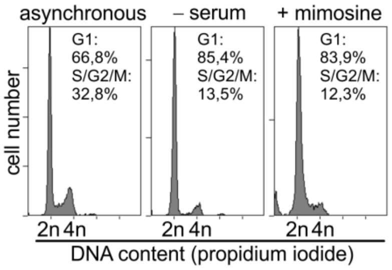

Fig S3. Cell cycle profiles of serum-starved or mimosine-treated QT6 cells

QT6 cells treated as described in Fig **3E-F** were stained with propidium iodide and analyzed by flow cytometry.

Fig.S4

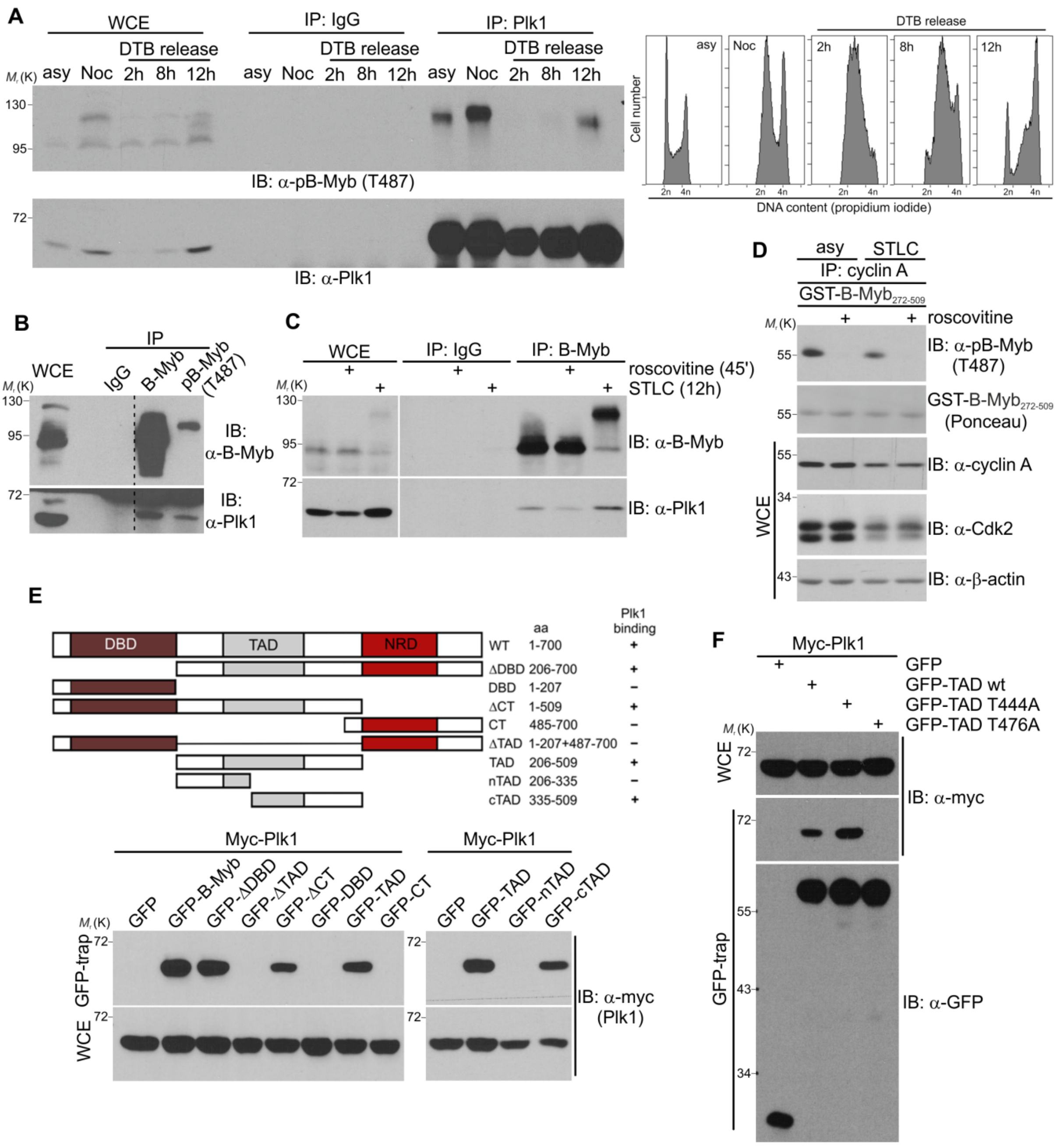

Fig S4. B-Myb interacts with Plk1 through the T476 phosphosite

A) Hek293 cells were released from double-thymidine block (DTB) for indicated times or treated with nocodazole for 8h or left untreated. *Left:* Whole cell extracts prepared from these

cells were immunoprecipitated with Plk1-specific or control IgG antibodies. Bound proteins and WCE were analyzed by WB as indicated. *Right:* Cell cycle distribution was analyzed by flow cytometry.

B) Whole cell extracts prepared from Hek293 cells were immunoprecipitated with pan-B-Myb (“brian”), pB-Myb(T487) or control IgG antibodies. Bound proteins and WCE were analyzed by WB as indicated.

C) Whole cell extracts prepared from Hek293 cells treated with roscovitine for 1h or with STLC for 12h were immunoprecipitated with B-Myb-specific or control IgG antibodies. Bound proteins and WCE were analyzed by WB as indicated.

D) In vitro kinase assay with recombinant GST-B-Myb (aa 272-509) fragment and cyclin A immunoprecipitates from untreated (asy) or STLC-treated Hek293. Roscovitine was added to the reaction mix as control of the assay specificity. Phosphorylation was monitored with antibodies against phosphorylated T487. β-actin was used as loading control.

E) *Top*: Schematic representation of GFP-B-Myb fragments used to map Plk1/B-Myb interaction. The numbers refer to amino acids of human B-Myb. *Bottom*: Lysates prepared from QT6 cells transiently expressing the indicated GFP-B-Myb proteins together with Myc-Plk1 were immunoprecipitated with GFP-trap beads. Immunoprecipitates and WCE were analyzed by WB with antibodies against the Myc-tag.

F) Lysates prepared from QT6 cells transiently expressing GFP, GFP-B-MybTAD(wt), GFP-B-MybTAD(T444A) or GFP-B-MybTAD(T476A) together with Myc-Plk1 were immunoprecipitated with GFP-trap beads. Immunoprecipitates and WCE were analyzed by WB with the indicated antibodies.

Fig.S5

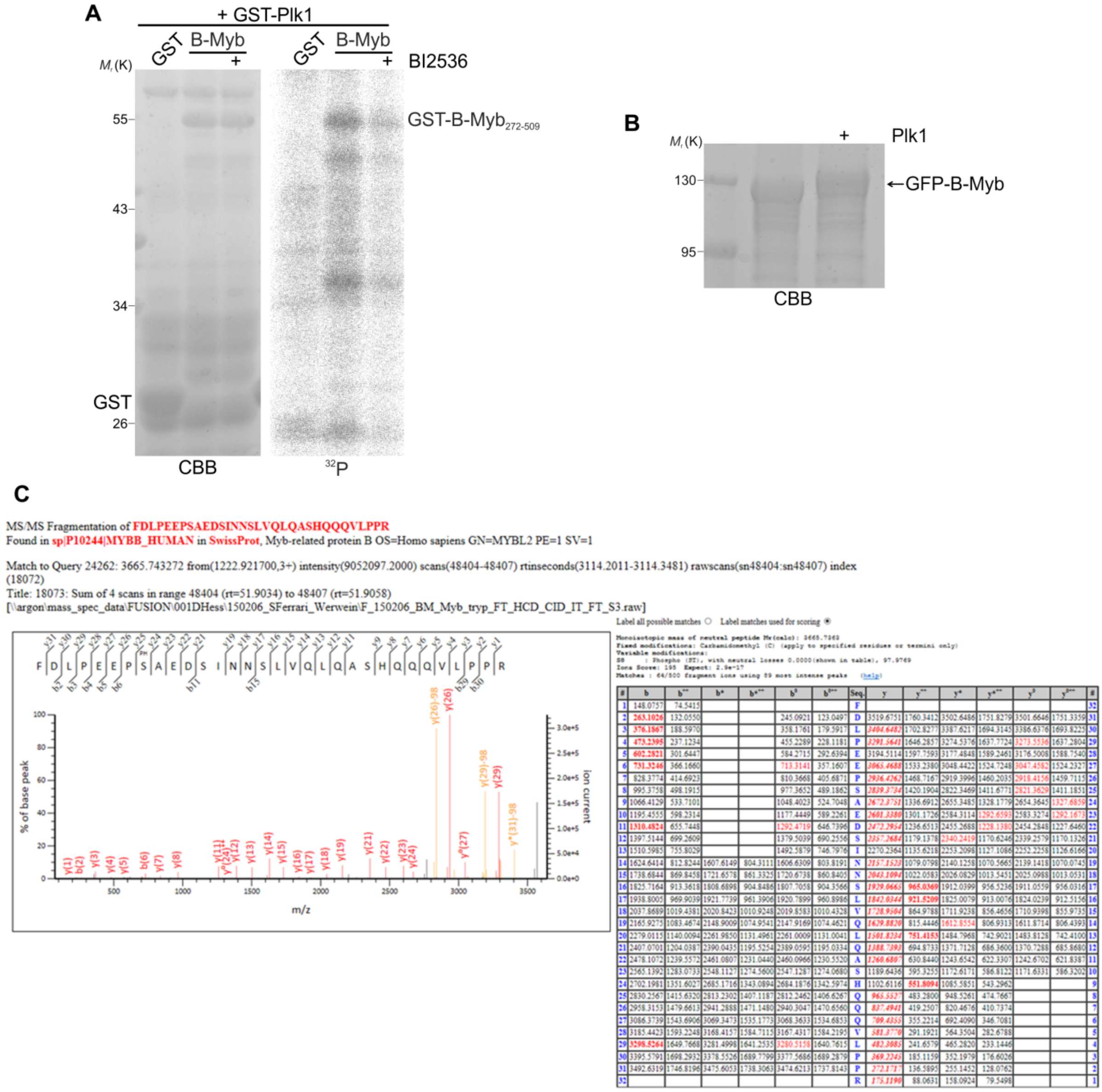

Fig S5. Identification of the Plk1 phosphorylation sites of B-Myb

A) In vitro protein kinase assay. GST-B-Myb (aa 272-509) was phosphorylated in vitro by recombinant GST-Plk1 with γ^{32}P-ATP, resolved by SDS-PAGE and visualized by autoradiography. A CBB-stained gel of the GST-proteins is shown on the left.

B) CBB-stained gel of immunopurified GFP-B-Myb expressed together with or without Plk1 in QT6 cells. Protein bands labeled with arrows were excised and analyzed by mass spectrometry.

C) Example spectra of a peptide with Ser335 phosphorylation identified with MASCOT. CID and HCD fragmentation spectra measured both in the orbitrap and in the iontrap of the same precursor of 1222.9217 (3+) were combined resulting in almost complete sequence coverage of 32 amino acid long peptide and an ion score of 195.

Fig.S6

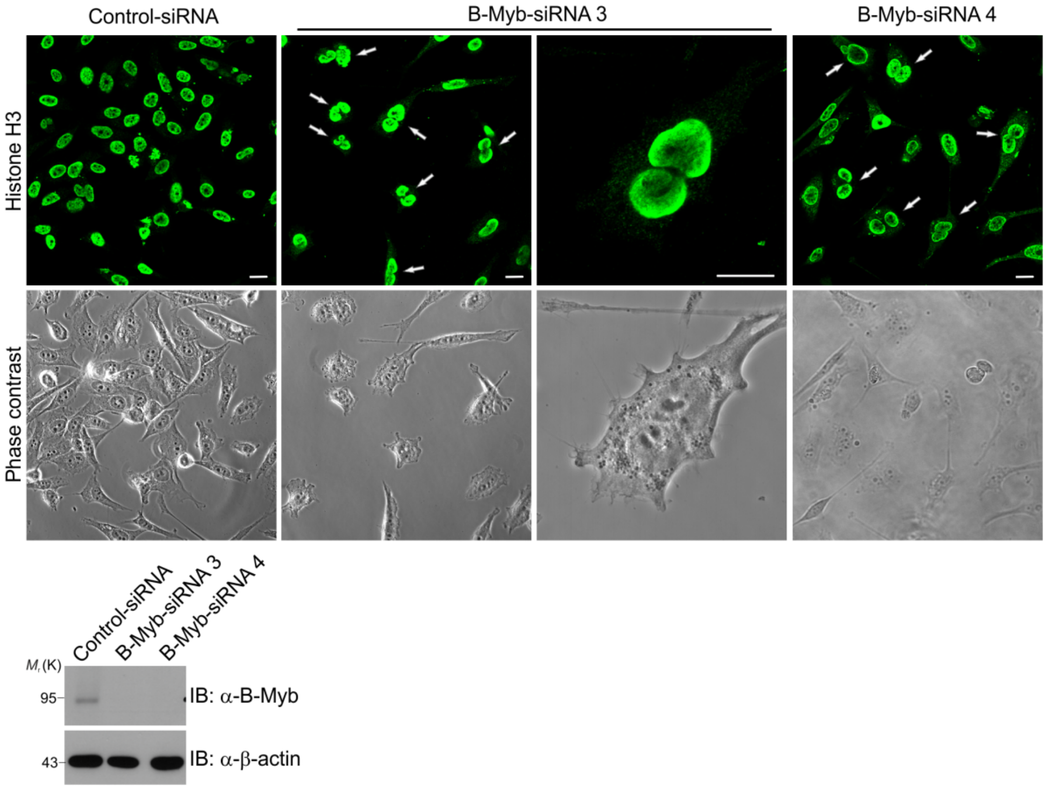

Fig S6. B-Myb knockdown in PC3 cells

PC3 cells were transfected with control or B-Myb-specific siRNAs, incubated for 48 h and analyzed by WB (*bottom*) or immunofluorescence (*top*). Nuclei were stained with anti-histone H3 antibody. Arrows indicate binucleated cells. Scale bars, 10 μm.

Fig.S7

Gene	meta-z score	DREAM target	MMB-FoxM1 target
BIRC5	12,2	yes	yes
FOXM1	12,05	yes	no
TOP2A	11,92	yes	yes
TPX2	11,72	yes	yes
CCNB1	11,41	yes	yes
NME1	11,38	no	no
CEP55	11,3	yes	yes
CENPF	11,18	yes	yes
CDKN3	11,04	yes	yes
TYMS	11	no	no
TRIP13	10,91	yes	no
CENPA	10,89	yes	yes
CDC20	10,85	yes	yes
BUB1	10,83	yes	yes
AURKA	10,77	yes	yes
POLD2	10,62	yes	no
TK1	10,6	yes	no
MCM2	10,31	yes	no
CCT3	10,27	no	no
MAD2L1	10,21	yes	yes
ENO1	10,18	no	no
AURKB	10,16	yes	yes
MYBL2	10,11	yes	no
DTL	10,07	yes	yes

NEK2	10,04	yes	yes
UCK2	9,98	no	no
RRM2	9,96	yes	yes
EXO1	9,95	yes	no
ADM	9,82	no	no
UBE2C	9,78	yes	yes
CHEK1	9,77	yes	no
MELK	9,77	yes	no
KIAA0101	9,77	yes	no
HJURP	9,64	yes	yes
KIF4A	9,63	yes	yes
RFC4	9,59	yes	no
KIF23	9,58	yes	yes
BUB1B	9,57	yes	yes
KIF14	9,56	yes	yes
PLK1	9,55	yes	yes
UBE2T	9,54	yes	yes
SLC16A1	9,52	yes	no
CKS2	9,52	yes	yes
CDT1	9,47	yes	no
HMMR	9,44	yes	yes
RNASEH2A	9,34	yes	no
GGH	9,33	yes	no
ESPL1	9,28	yes	yes
CDC25C	9,23	yes	yes
CDCA5	9,19	yes	yes

Fig S7. List of Top 50 pan-cancer genes

50 genes with the highest z-score were obtained from the PRECOG database (https://precog.stanford.edu/) and classified as targets of DREAM or MMB-FoxM1 complexes (http://www.targetgenereg.org/).
